# Alteration in expression and subcellular localization of the androgen receptor- regulated FAM111A protease is associated with emergence of castration resistant prostate cancer

**DOI:** 10.1016/j.neo.2025.101181

**Published:** 2025-05-29

**Authors:** Maria Malvina Tsamouri, Stephen J. Libertini, Salma Siddiqui, Maitreyee K. Jathal, Blythe P. Durbin-Johnson, Clifford G. Tepper, Eva Corey, Jun Luo, Kenneth A. Iczkowski, Paramita M. Ghosh, Maria Mudryj

**Affiliations:** a Department of Urologic Surgery, Sacramento, CA 95817, USA; b Department of Medical Microbiology and Immunology, Davis, CA 95616, USA; c Veterans Affairs-Northern California Health Care System, Mather, CA 95655, USA; d Division of Biostatistics, Department of Public Health Sciences; e Department of Biochemistry and Molecular Medicine, University of California, Davis, Davis, CA 95616, USA; f GU Cancer Research Lab, Department of Urology, University of Washington, Seattle, WA 98195, USA; g Department of Urology, James Buchanan Brady Urological Institute, School of Medicine, Johns Hopkins University, Baltimore, MD, USA; h Department of Pathology and Laboratory Medicine, University of California Davis, Davis-Health, CA 95817, USA

**Keywords:** FAM111A, Androgen receptor, Prostate cancer, Castration resistant prostate cancer, Metastases, Nucleoli, Subcellular

## Abstract

The androgen receptor (AR) is a pivotal regulator of growth and survival of prostate cancer (PCa) and the majority of lethal castration-resistant prostate cancers (CRPC) remain reliant on AR signaling. PCa exhibits variability in progression and responses to treatment suggesting genetic heterogeneity. Two independent studies identified PCa predisposing single nucleotide polymorphisms (SNPs) within the FAM111A protease gene, but the mechanistic basis of this association remained elusive. Our *in vitro* and *in vivo* studies uncovered that AR represses FAM111A in castration sensitive and resistant cells via an AR binding site within the *FAM111A* gene. FAM111A levels are significantly lower in matched castration-resistant than in castration-sensitive cells and xenografts, and lower in metastatic lesions than in primary tumors. We discovered that FAM111A is AR-repressed in castration sensitive PCa xenograft and multiple PCa cells. Additionally, FAM111A subcellular localization changes dramatically with acquisition of castration resistance, where in castration sensitive cells FAM111A is predominantly in the nucleoli, but with castration resistance it becomes more dispersed in the nucleus and in the cytoplasm. FAM111A depletion in castration sensitive and resistant cells enhances the efficacy of PARP1 inhibitors olaparib and niraparib, consistent with its role in DNA repair. Moreover, FAM111A depletion reduces AR target gene prostate specific antigen (*PSA)* and transmembrane serine protease 2 (*TMPRSS2)* transcription, indicating that FAM111A modulates AR-dependent gene expression forming a FAM111A-AR co-regulatory loop in PCa. Our studies argue that AR-dependent FAM111A regulation modulates PCa gene expression, acquisition of castration resistance, and sensitivity to agents that target DNA damage repair.

## Highlights

• The AR represses FAM111A protease transcription in multiple castration sensitive and resistant prostate cancer cells.
• Metastatic lesions exhibit decreased FAM111A expression when compared to primary PCa tumors.
• FAM111A subcellular localization is restricted to nucleoli in castration sensitive cells but becomes progressively redistributed to the nuclear and cytoplasmic compartments with acquisition of castration resistance.
• Depletion of FAM111A sensitizes castration sensitive and resistant cells to PARP inhibitors.
• Expression of AR-regulated transcripts decreases on FAM111A depletion indicative of an AR-FAM111A regulatory loop.

## Introduction

PCa remains the most commonly diagnosed cancer in men in the United States [1]. PCa is initially treated by androgen deprivation therapy (ADT) due to its dependence on AR signaling. Progression to CRPC that is refractory to the standard ADT strategy continues to be a great challenge [[2], [3], [4]]. PCa exhibits great heterogeneity, and multiple mechanisms drive progression of hormone-sensitive PCa to CRPC, but they center on retaining AR signaling, although the mechanistic details and the AR transcriptional output varies [[5], [6], [7], [8], [9]].

Prominent risk factors for the development of PCa include age, positive family history and ancestry. Genome wide association studies (GWAS) have identified over 200 SNPs associated with increased risk of developing PCa, or an aggressive form of the disease [10]. Two studies identified SNPs within the *FAM111A* trypsin-like protease gene that predispose to PCa. A GWAS of PCa susceptibility in a Japanese population identified a SNP in the third intron of the *FAM111A* gene that was significantly associated with PCa development [11]. A subsequent GWAS study of familial PCa identified two distinct predisposing variants, which mapped to the protein coding exon [12], together arguing that *FAM111A* may have a role in PCa. But the function of FAM111A in PCa has remained elusive.

FAM111A has been implicated in other disease processes [13,14]. Activating dominant mutations in FAM111A have been identified as the cause of the rare genetic disorders Kenny Caffey Syndrome Type 2, gracile bone dysplasia, and osteocraniostenosis characterized by impaired skeletal development, short stature, hypoparathyroidism, electrolyte disturbances, dental and ocular abnormalities, and seizures and spasms [[15], [16], [17]]. FAM111A mutations have been associated with hypomagnesemia [18]. FAM111A is inhibitory for SV40, pox, and ZIKA virus replication. SV40 studies identified FAM111A as a host factor that limits virus production [19]. Similarly, deletion mutations of the vaccinia and rabbitpox serine protease inhibitor 1 can be rescued by depletion of FAM111A [20] which inhibits vaccinia replication by degrading the viral protein I3 [21]. ZIKA infection leads to an increase of FAM111A which serves to limit viral replication [22]. Thus, FAM111A serves to limit viral life cycles of three unrelated viruses.

Of the five exons that constitute the *FAM111A* gene only two encode the FAM111A protein. The ∼70 KD protein includes an N-terminal proliferating cell nuclear antigen (PCNA) binding PIP domain, an adjacent ssDNA binding region, and a C-terminal trypsin-like protease domain. An autoproteolytic site maps between the catalytic domain and the ssDNA binding region. FAM111A interacts with PCNA at nascent replication sites through its N-terminal PIP domain to promote DNA replication [23]. FAM111A interaction with ssDNA is reliant on the ssDNA binding domain [24], and its protease function is required for resolving replication fork-blocking DNA-protein complexes [25,26]. Hence, FAM111A depletion potentiates the activity of PARP1 and topoisomerase inhibitors, agents that prevent DNA repair to promote cell death [25]. However, FAM111A is not essential for replication in all cellular contexts, since cells deleted for FAM111A are able to proliferate proficiently [25], and *FAM111A* knockout mice are viable and do not exhibit any overt phenotypic abnormalities [27]. Lastly, FAM111A has been implicated in several malignancies including gastric cancer, cervical cancer, and diffuse lower-grade glioma [[28], [29], [30]].

Here we report the reciprocal regulation of FAM111A and AR in PCa cells. FAM111A is AR repressed in multiple castration-sensitive and castration-resistant cells *in vitro* and *in vivo*, FAM111A levels are lower in castration-resistant cells and lower in metastatic lesions than in primary tumors. Moreover, studies of multiple castration-sensitive and resistant cells revealed changes in FAM111A localization with emergence of castration resistance. Depletion of FAM111A sensitizes cells to PARP1 inhibitors olaparib and niraparib. Additionally, FAM111A depletion reduces expression of AR target genes *KLK3/PSA* and *TMPRSS2*, indicating FAM111A and AR constitute a regulatory loop.

## Material and methods

### Characteristics of prostate tumor microarrays

All human tissues were collected with approval from the Institutional Review Board (IRB), at Northern California Health Care System (VANCHCS) and all experiments were performed in accordance with VANCHCS IRB guidelines and regulations. All patients provided informed consent to participate under the IRB-approved protocol. Tissue microarrays (TMA) representing a total of 78 patients who underwent radical retropubic prostatectomy between 1999 and 2004 were obtained in a deidentified manner from the VANCHCS biorepository that collected the tissues, and clinical data were extracted from the VANCHCS archives. Patient characteristics have been previously described [31].

### Databases

The following databases were used in the study: cBioPortal, ToppGene, EnrichR, and GEO Profiles.

### Immunohistochemistry

Immunohistochemical studies were conducted as previously described [32]. The following antibodies were used: Ki67 (MIB-1, DAKO), AR (sc-816, Santa Cruz Biotechnology), FAM111A (NBP1-82103, Novus). The degree of staining was evaluated in a semiquantitative fashion by S.S. noting both the intensity of staining as well as the percentage of cells exhibiting that intensity in the nucleus and the cytoplasm. The intensity of the staining was scored as no staining (0), weak (1), intermediate (2), and strong (3) staining. The composite score was the percent of cells staining multiplied by the intensity of staining. This was averaged for the cores from the same tumor.

### Cell culture and transfection of siRNA

LNCaP (CRL-1740), C4 (CRL-3313), and C4-2 (CRL-3314), and CWR22Rv1 (22Rv1) (CRL-2505) cells were obtained from American Type Culture Collection (ATCC) and used under passage 20. LNCaP AI cells were generated in our laboratory by continuous culture of LNCaP cells in steroid depleted media [27]. Cells were propagated in RPMI 1640 supplemented with 10 % fetal bovine serum (FBS) or charcoal stripped FBS (CCS), 2 mmol/L L-glutamine, 100 U/ml penicillin, and 100 mg/ml streptomycin (Invitrogen) at 37°C and 5 % CO_2_. 3 × 10^5^ cells were plated in 60 mm dishes and transfected 24 h later using with Lipofectamine 2000 or RNAi Max (Invitrogen) and 100 nM siRNA obtained from Dharmacon (currently Horizon) FAM111A siRNA: GGUCAAUGUGUAAGGGUGA (J-013926-12), AR siRNA smart pool (L003400-00), control non-targeting oligonucleotide (D-001210–01). The transfection mix was removed 24 h later and replaced with normal growth medium. Cells were harvested 72 h post-transfection. Olaparib and niraparib were obtained from Selleckchem.

### Immunofluorescence (IF)

Cells were plated at 60,000 cells per well in a 2 chambered glass slide. The following day, the chambers were removed, and the slides were rinsed with pre-warmed 1x PBS, followed by 4 % paraformaldehyde fixation, washed in PBS + 0.3 % Triton-X100 (PBS-TX) and incubated in PBS-TX + 1 % BSA, and NOPP140 antibodies (Santa Cruz sc-374033), diluted 1:500 in PBS-TX +1 % BSA. Slides were treated overnight at 4C. Slides were washed in PBS-TX, followed by the secondary antibody (Alexa Fluor 488, #A11008 and Alexa Fluor 647 #A21235, Invitrogen) for 1 h at room temperature (in the dark). Vectashield with DAPI was applied and allowed to dry before visualization using an EVOS FL2 Auto Imaging System using 40X magnification. Captured images were transferred and processed using standard protocols to generate merged overlays with the ImageJ software (https://imagej.net/ij/).

### Western immunoblot analysis

Following washes with cold PBS, cells were directly placed in a radioimmunoprecipitation assay lysis buffer that contained a protease inhibitor cocktail (Sigma). Thirty micrograms of protein were separated on 8 %, 10 %, or 12 % SDS–PAGE gels and transferred to 0.22 mm GE Nitrocellulose supported membrane (BioExpress). The membrane was blocked with 5 % nonfat dry milk in PBS and 0.1 % Tween-20 (PBS-T), or OneBlock Western-CL blocking buffer (Genesee Scientific; El Cajon, CA). The following antibodies were used: from Santa Cruz Biotechnology: AR (sc-816), GAPDH (sc- 32233), from Novus: FAM111A (NBP1-82103), from Cell Signaling: tubulin (2125). The following day, membranes were washed with PBS-T 3 times, incubated with secondary antibody conjugated to HRP, and development was carried out using SuperSignal West Femto chemoluminescence (Thermo Scientific) or imaged using LI-COR near-infrared western blot detection. Gel loading was standardized either by GAPDH or tubulin. ImageJ software was used to quantify proteins standardizing to the loading control.

### RNA preparation and realtime PCR

Studies were conducted as previously described [33].Total cellular RNA was prepared utilizing RNeasy Mini kit (Qiagen, Inc. CA) and cDNA was synthesized from RNA using the qScript XLT cDNA SuperMix ((#95161, Quanta Biosciences). Realtime PCR was conducted using the PerfeCTa SYBR Green FastMix Low ROX kit (#95074 Quanta Biosciences). PCR conditions had initial denaturation step at 95 C for 2 min 30 s, 40 cycles at 95 C for 13 s. HPRT1 was used as the endogenous expression standard. Data was collected with ViiA 7 (Applied Biosystems) and analyzed using the efficiency corrected relative standard curve method. Primers are described in supplementary data.

### Library preparation and RNA-Sequencing

RNA-Sequencing (RNA-Seq) libraries were prepared from 1 μg total RNA using the NuGen ovation RNAseq system V2 (currently TECAN, Redwood City, CA) according to the manufacturer’s protocol. Poly-adenylated mRNA was purified from total RNA and ribosomal RNA removed by two rounds of binding to magnetic oligo-dT beads. RNA purity and concentration were measured using the NanoPhotometer Pearl (Implen). RNA-Seq libraries were prepared and sequenced on a lllumina NovaSeq 6000 System (2 × 150 bp, paired end). Data analysis was performed with a TopHat2-Cufflinks-Cuffdiff [34,35] pipeline for mapping/alignment of raw sequence reads (FASTQ format) to the reference human genome assembly (GRCh37/hg19), transcript assembly, and quantitation of gene and transcript expression as FPKM (Fragments Per Kilobase of transcript per Million mapped reads). Data were annotated for unique genes/transcripts with Ensembl Release 82 (GRCh37/hg19). Group comparisons were conducted with Cuffdiff, and genes meeting a statistical threshold of an adjusted *P*-value < 0.05 were assigned as differentially expressed. Log2(fold change) and adjusted *P*-values were used as input for volcano plots, and normalized FPKM values utilized for hierarchical clustering and heatmap visualization. The ToppGene Suite18 and EnrichR19 platforms defined enrichment for molecular functions, biological processes, cellular components, and pathway alterations.

### Chromatin immunoprecipitation

ChiP studies were conducted as previously described [31]. Magna ChIP™ HiSens Chromatin Immunoprecipitation Kit (Millipore) was used the following modifications: 3 μg of normal rabbit IgG (Santa Cruz Biotechnology) was added to the lysate for 1.5 h at 4°C and precleared with 40 μl of protein A/G magnetic beads cells at room temperature. Precleared chromatin was split into aliquots: 1.5 μg of ChIPAb + Androgen Receptor- ChIP Validated Antibody (Millipore) or rabbit IgG (Santa Cruz Biotechnology) were added to aliquots. Following cleanup, the DNA was fractionated on a 1 % low melt agarose gel and 200 and 500 bp fragments were excised and purified using the QIAquick gel extraction kit (Qiagen, Inc.). DNA products were quantified by qPCR using primer sets (Supplementary Data) using PerfeCTa SYBR Green FastMix Low ROX according to manufacturer's recommendations (Quanta Biosciences, Gaithersburg, MD). Recovered products after ChIP were normalized to the respective negative control (IgG) using the formula ΔCt = Ct target product or input – Ct IgG, and further calculated as percent of input.

### Animal studies

The study was conducted under an approved IACUC protocol. Nu/nu athymic 6-8 week old male mice were obtained from Harlan Sprague Dawley, Inc. (Indianapolis, IN) and acclimated for a week. The mice were fed standard chow and water ad libitum, and they were maintained under a 12 h light, 12-hour dark cycle. Mice were implanted subcutaneously with sustained release testosterone pellets (12.5 mg, 90-day release; Innovative Research of America). Suspensions of castration sensitive CWR22 PDX cells were made in a 1:1 solution of RPMI 1640 (GIBCO) and Matrigel (BD Biosciences). Xenografts were established by subcutaneous injections of 2.5 × 10^6^ cells in 100ul/injection into the flank. When palpable tumors were observed, animals were block randomized based on tumor size into two groups: (i) left intact (sham operated) or (ii) castrated by bilateral orchiectomy and removal of the testosterone pellet. Tumor volume (length X width^2^ X 0.5) and animal body weight were recorded twice weekly. The following were humane endpoints for euthanasia: i) evident clinical signs of distress, ii) weight loss of >20 %, iii) tumor volume of >1,500-2,000 mm3, iv) tumor length of >20 mm in any direction. The animals were followed for approximately four weeks, then euthanized and the tumors were resected. Half of each tumor was formalin fixed and half was frozen in liquid nitrogen. The propagation of LuCaP PDX tumors was conducted as previously described [36]. Castration sensitive tumors were propagated in intact male mice, while castration resistant tumors were propagated in castrated male mice.

### Treatment with PARP inhibitors- Cell viability assay

LNCaP or 22Rv1 cells were plated in 24-well plates (30,000 cells/well) and transfected 24h later with either FAM111A siRNA (siFAM111A, ON-TARGETplus, SMARTPool # L-013926-01-0010, Dharmacon) or control siRNA (siC) using jetPRIME transfection reagent (Polyplus) per the manufacturer’s instructions. After 24 hours, medium was replaced with olaparib, niraparib or equal concentration of vehicle control (DMSO), as indicated, and cells were treated in quadruplicates. The appropriate doses for each compound and cell line were defined from dose titration experiments. The cells were treated for 72 hours, and cell viability was measured using Cell Counting Kit-8 (CCK-8) per the manufacturer’s instructions (Dojindo; Rockville, MD).

### Human prostate tissue samples

All tissues were obtained under a Johns Hopkins School of Medicine approved Institutional Review Board protocol and RNA and cDNA preparation were conducted as previously described [37].

### Statistical analysis

Clinical Information was compared between patients with BCR (Biochemical Recurrent) and no-BCR cancer using Wald Chi-square statistics. Median staining levels were compared between tumor and non-tumor areas from the same subject using Mann-Whitney tests. TMA immunohistochemistry (IHC) data were summarized across positions by taking the median of non-missing values. The correlations between staining levels and demographic characteristics were estimated using Pearons Correlation. Survival analyses used Weibull models and was conducted using SAS version 9.3. Mouse tumor data were analyzed by normalization of all measurements to pre-operation (sham or castration) measurements for each individual mouse, then mean and standard errors calculated for the aggregate group. Graphs were generated using Excel or SigmaPlot 12.0. All (real time) RT PCR analyses of mRNA levels were conducted in triplicate. For RT-qPCR experiments, the delta-delta Ct method was used to calculate fold change in gene expression. A p < 0.05 was considered as statistically significant.

## Results

### Cellular context governs the AR-regulated transcriptome

**Fig. 1:**
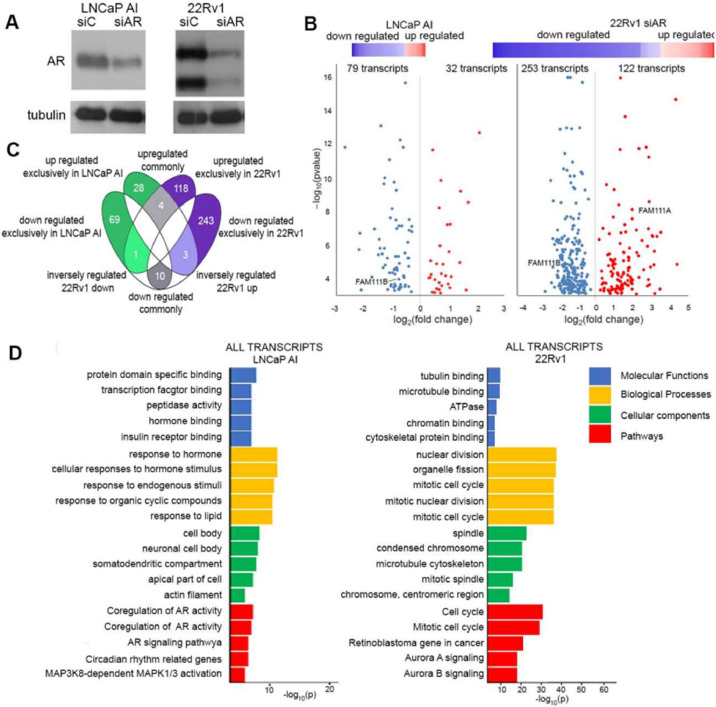
**Transcriptome changes on AR depletion in 22Rv1 and LNCaP-AI cells. A**. Immunoblot analysis confirmed that AR targeting siRNA effectively reduced expression of both AR isoforms (top panel). **B.** A heat map (top) and a volcano plot (bottom) indicate that more transcripts were repressed than activated following AR depletion. **C.** Comparison of transcript regulation on AR depletion in LNCaP-AI and 22Rv1 cells. Transcripts were divided into those exclusively altered in LNCaP-AI (green) or 22Rv1 cells. (purple). Transcripts commonly altered in both models are shown in grey. **D.** Gene enrichment analysis of the identified transcripts for each cell model. Molecular function (blue), biological processes (yellow) cellular components (green) and pathways (red) altered in AR depleted cells. Transcriptome data available in Geo (GSE286417).

AR activity at castrate levels of androgen is sufficient to regulate the expression of genes that are required for proliferation and survival of castration resistant 22Rv1 cells which exhibit decreased proliferation on AR depletion [38] and LNCaP-AI which express AR, but do not exhibit changes in proliferation on AR depletion [39]. To define AR-dependent transcriptomes in these cells, AR signaling was reduced by AR-targeting siRNA (siAR). Non-targeting siRNA (siC) served as a control (Fig. 1A). Following enrichment of polyadenylated transcripts, RNA-seq studies identified siAR-dependent alterations in gene expression. Volcano plots (Fig 1B), show that there are twice as many repressed as elevated transcripts in both cellular contexts. In 22Rv1 cells, 253 transcripts are decreased and 122 are elevated, while in LNCaP-AI cells 79 transcripts are decreased and 32 are elevated (Supplementary Data 1). Comparison of transcriptome changes shows that they are mostly distinct (Fig 1C), where only ten transcripts, AR, ASPH, FAM111B, PCGF5, LBR, SLC45A3, PPAP2A, NKX3.1, PMEPA1, TMEF2 are commonly down regulated and four, APP, BCHE, SI, and MAP2 are upregulated (Fig. 1C). Previous reports have shown that NKX3.1, SLC45A3, PMEPA1, APP, BCHE are either direct AR targets or AR-regulated genes [[40], [41], [42], [43]]. Gene enrichment analysis (GEA) using the ToppGene Suite [44] uncovered that in 22Rv1 cells the five most altered molecular functions are tubulin and microtubulin binding, ATPase, chromatin binding and cytoskeletal binding and the most altered biological processes all center on the mitotic cell cycle, and nuclear division. Altered cellular components are spindle, condensed chromosome, and cytoskeleton. These changes lead to cell cycle, retinoblastoma, Aurora A and B signaling pathway alterations indicative of a G2/M checkpoint blockade (Fig. 1D).

In contrast, molecular functions altered on AR depletion in LNCaP-AI cells are protein specific binding, transcription factor binding, peptidase activity, and hormone binding. Altered biological processes are responses to hormone, endogenous stimuli, and organic compounds, while altered cellular components are cell body, neuronal cell body, somatodendritic compartment, and apical part of cells. Pathway changes center on AR activity, circadian rhythm, and MAPK1/3 activation. Known AR targets KLK2 and 3, PCA3, PMEPA1, SLC45A3 and NKX3.1 are AR regulated; however, AR does not regulate transcription of cell proliferation or survival genes, reinforcing that in these cells the connection between AR and proliferation no longer exists, but AR retains transcriptional control of a distinct gene cohort (Supplementary Data). In PCa the AR regulates several proteases including KLK2 and 3 and TMPRSS2 which are direct AR target genes. This analysis uncovered additional proteases, FAM111A and B, that are AR regulated in PCa cells but their role in this malignancy is unknown.

### Paralogue genes FAM111A and FAM111B are AR-regulated in a reciprocal manner

**Fig. 2:**
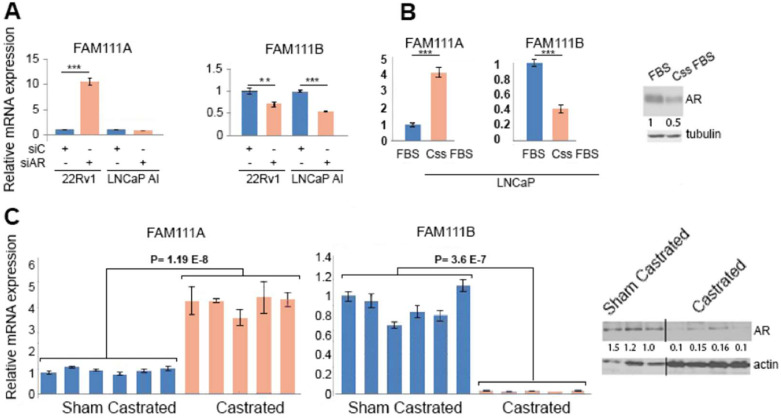
**FAM111A and B are reciprocally regulated in PCa. A.** FAM111A transcript levels are elevated on AR depletion in 22Rv1, but not LNCaP-AI cells while FAM111B is decreased in both cells on AR depletion. **B.** LNCaP cells cultured in the presence or absence of steroids. AR protein expression was normalized to the loading control. **C.** CWR22 xenografts in castrated and sham castrated male mice confirm reciprocal AR-dependent regulation of FAM111A and B (left panel). qRT-PCR verified transcripts alterations. All qRT- PCR analysis was conducted in triplicate using the delta-delta Ct method to calculate fold change in gene expression. *P-value < 0.05. ** P ≤ 0.005, *** P ≤ 0.0005. Reduced AR protein expression in castrated animals (right panel). AR expression in castrated and sham castrated tumors was normalized to loading control.

Two independent GWAS identified PCa predisposing SNPs within the FAM111A gene, but little is known about their regulation in PCa cells. Reciprocal regulation of FAM111A and FAM111B on AR depletion in 22Rv1 cells and FAM111B repression in LNCaP-AI following AR knockdown was validated using qRT-PCR (Fig. 2A). AR-dependent regulation of these genes was further investigated in hormone sensitive LNCaP cells and CWR22 xenografts. In steroid depleted media castration-sensitive LNCaP exhibit reduced AR levels, a marked increase of FAM111A and decrease in FAM111B (Fig. 2B), hence unlike in LNCaP-AI cells, decreased AR signaling in the parental LNCaP cells results in increased FAM111A expression, reinforcing the results evident in 22Rv1 cells.

In CWR22 xenograft model FAM111A levels are low when tumors are propagated in intact animals but are greatly increased following castration when AR signaling is inhibited (Fig. 2C). FAM111B is regulated in an inverse manner. Hence, FAM111A is AR repressed and FAM111B is AR induced in multiple PCa models. Since FAM111A GWAS studies identified SNPs within the FAM111A gene associated with increased risk for PCa development [11,12], we focused on this gene.

### FAM111A expression is decreased in metastatic lesions

**Fig. 3:**
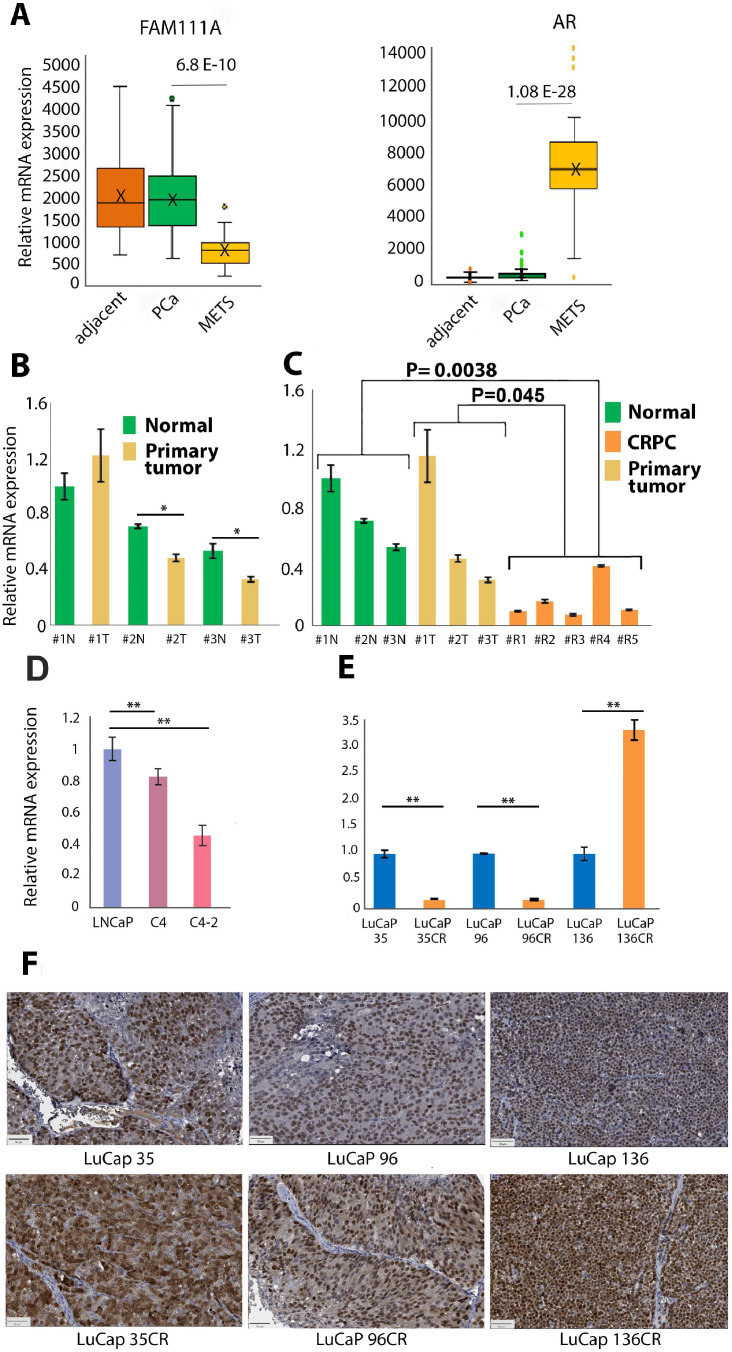
**FAM111A levels are repressed in castrate resistant cells. A.** Geo profiles (GSE6919) analysis of normal, adjacent, primary PCa and metastatic tissues (MET) identifies a significant decline of FAM111A and elevation of AR in metastatic lesions. **B.** Primary PCa and matched normal tissue indicate that FAM111A is reduced in tumors. **C.** Comparison of normal prostate tissue, primary tumors with CRPC indicates a statistically significant decrease of FAM111A transcription in CRPC tumors. **D.** FAM111A levels in LNCaP-C4-C4-2 progression model. **E.** FAM111A levels in matched sets of castration sensitive and resistant LuCaP tumor cells. qRT-PCR verified transcripts alterations. All qRT- PCR analysis was conducted in triplicate using the delta-delta Ct method to calculate fold change in gene expression. * P ≤ 0.05, ** P ≤ 0.005. **F.** IHC staining shows AR expression in castration resistant and castration sensitive LuCaP tumors.

Transcriptional studies that assessed gene expression in primary PCa, tumor-adjacent tissue and metastatic lesions Gene Expression Omnibus (GEO) Accession GSE6919 revealed that FAM111A expression is higher in primary PCa and tumor-adjacent tissue than in metastatic lesions (Fig. 3A). Conversely, AR expression in metastatic lesions is greatly elevated in comparison to primary tumors as previously reported [11], further arguing that FAM111A and AR expression is negatively associated.

FAM111A mRNA expression was evaluated in normal human prostate tissue and in primary PCa and metastatic CRPC (mCRPC) lesions. In matched tumor and adjacent tissue, in two of the three samples there is a statistically significant decrease of FAM111A in the tumor tissue (Figure 3B). A comparison of FAM111A in normal and mCRPC sample, and primary tumors and mCRPC indicates that FAM111A transcription is significantly decreased in the mCRPC (Fig. C).

The LNCaP progression model of castration resistance, where C4 and C4-2 cells are progressively more castration resistant than the parental LNCaP has been well studied [45]. While C4 and C4-2 cells proliferate in androgen depleted conditions, they retain AR expression and exhibit high expression of PSA, a known AR target gene. FAM111A levels are highest in LNCaP cells, decrease in C4 cells, and are lowest in C4-2 cells (Fig. 3D).

FAM111A expression was assessed in three matched sets of castration-sensitive and resistant-LuCaP PDX models [36]. In two matched tumor sets LuCaP 35/35CR and LuCaP 96/96CR FAM111A levels are significantly higher in the castration-sensitive than in the resistant tumors. In the LuCaP136/136CR set, we observed the opposite, where FAM111A levels were greater in 136CR tumors (Fig. 3E). IHC analysis shows AR expression in castration sensitive and resistant LuCaP tumors, where AR nuclear staining is elevated in LuCaP136CR tumors (Fig. 3F).

### FAM111A is a direct AR target

**Fig. 4:**
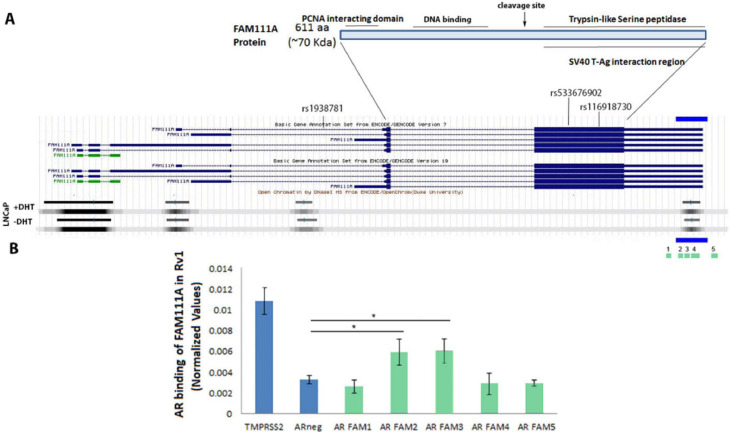
**The AR binds to the 3′non-translated region of the FAM111A gene. A.** Schematic of the AR gene. An identified AR binding site maps to the 3′ region of the gene (blue bar). DNase sensitive sites in LNCaP cells in the presence an absence of androgens map to distinct regions of the FAM111A gene including an overlap with the AR binding domain (top bars). The green bars denote sequences (1-5) that were analyzed in the AR-ChIP study. **B.** ChIP studies indicate that the AR binds to region 2 and 3 of the identified AR binding domain. The AR binding site in TMPRSS2 served as a positive control, while an unrelated region mapping near the p4ARF gene served as a negative control. Recovered products after ChIP were normalized to the respective negative control (IgG) using the formula ΔCt = Ct target product or input – Ct IgG, and further calculated as a percentage of input. * P ≤ 0.05.

PCa predisposing SNPs localized to the FAM111A gene, but there is little information about the regulation of FAM111A. Using published data, we found that an AR-binding region maps to the 3′ non-translated region of FAM111A [Fig. 4A, Supplementary Data) [46]. This region does not contain a canonical androgen response element (ARE) or half site, but an analysis of the publicly available ENCODE data found that this region is adjacent to GATA binding sites [47]. ENCODE database analysis also identified FAM111A DNase sensitive regions in LNCaP cells cultured in the presence and absence of steroids. A very strong DNase sensitive site maps to the canonical transcriptional start site, and a weaker site at what appears to be a second transcriptional start site. A DNase sensitive site overlaps the identified AR-binding region. Notably, of the 77 cell lines and tissues available and analyzed on the ENCODE database, LNCaP cells are the only prostate cells in this cohort and the only cells that harbor a DNase sensitive site that overlaps this AR binding region.

ChIP studies confirmed AR binds to this region, where the TMPRSS2 AR-binding site served as a positive control. A series of primer sets corresponding to sequences surrounding and spanning the putative AR-binding region were used in the study. Our ChiP analysis refined the previously identified AR-binding site to a region spanning 213 nucleotides within the 3′ non-coding region of FAM111A (Fig. 4B, Supplementary Data 2). These results strongly suggest that this gene is a direct AR target.

### FAM111A subcellular localization in PCa differs in castration sensitive and resistant cells

Archival PCa tissues assembled into a 78-sample array were evaluated for FAM111A expression. Eight of the samples were pathological stage T2a, one was T2b, 50 were T2c, 13 were T3a and six were T3c (Table 1), while Gleason grade ranged from 5 to 9. FAM111A nuclear and cytoplasmic localization was scored as no staining (0), weak (1), intermediate (2), and strong (3) staining. The composite score was the percent of cells staining multiplied by the intensity of staining. This was averaged for the cores from the same tumor.

**Table 1:** Characteristics of 78 patients with primary (localized) PCa, who underwent radical retropubic prostatectomy from 1999-2002.

Number of patients Age range Race		78
		41-74
	African Americans	19
	Asian Americans	1
	Caucasians	51
	Hispanic	5
	Native Americans	1
Surgical Gleason score	5 and 6	36
	7	33
	8 and 9	9
Pathological stage	T2aNxMx	8
	T2bNxMx	1
	T2cNxMx	50
	T3a	13
	T3c	6

**Fig. 5:**
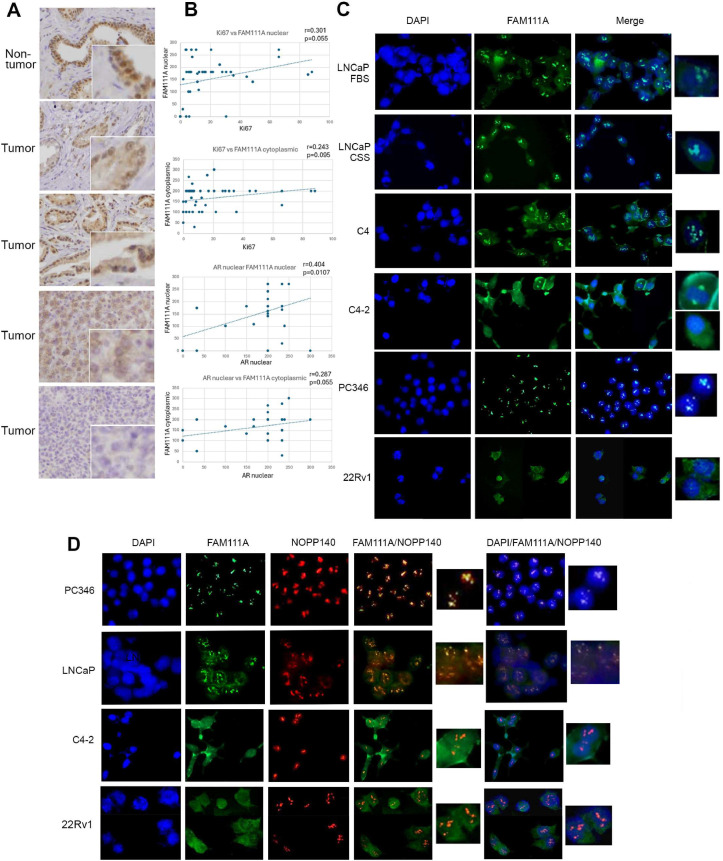
**FAM111A levels and subcellular localization differ in castration sensitive and resistant cells. A.** Characterization of FAM111A protein in PCa tumor and non-tumor tissue. **B.** Correlation of FAM111A staining with AR and Ki67 staining. R= correlation coefficient, P= statistical significance. **C.** IF studies in various PCa cells identifies alterations of FAM111A subcellular localization in castration sensitive and resistant cells. **D.** Co-staining with nucleoli protein marker NOPP140 indicates that FAM111A localizes to nucleoli in castration sensitive, but not in castration resistant cells.

In non-tumor cells FAM111A is detected in the nucleus and cytoplasm, and more intense staining foci are apparent in the nucleus, suggesting nucleoli staining. FAM111A staining in tumor cells varies, but in some tumors, staining is nuclear and cytoplasmic with intense nuclear foci. In other tumors staining is more cytoplasmic with more intense staining at the nuclear periphery, and in some tumors FAM111A staining is either very weak or absent (Fig. 5A).

Tumors were also stained with Ki67 to detect proliferating cells. Ki67 staining is restricted to the nucleus and as expected, there is a significant increase in Ki67 levels in tumor cells when compared to adjacent non-tumor cells (p=8.9 E-07). In cancer cells there is a significant correlation between nuclear AR and nuclear FAM111A staining (p=0.0107) (Figure 5B), and a trend of negative association between nuclear AR and cytoplasmic FAM111A (P=0.0556), and between Ki67 and nuclear FAM111A (p= 0.0559), but these trends are not evident in adjacent non-tumor cells. Additionally, in tumor cells there is a correlation between PSA failure and cytoplasmic FAM111A (p=0.0406), nuclear AR and pathological stage (p=0.0302), and a trend between PSA failure and pathological stage (p=0.065). There was a trend between preoperative PSA and Ki67 and preoperative PSA and pathological stage (Table 2).

**Table 2:** Correlations between tumor characteristics were calculated using Pearson correlation coefficient. Statistically significant correlations are bold, correlations trending towards significance are underlined.

	Ki67	AR (nuclear)	AR (cytoplasmic)	FAM111a (nuclear)	FAM111a (cytoplasmic)	PSA failure	surgical Gleason Grade	Pathological stage	Pre-op PSA
Ki67		r=0.212; p=0.088	r=-0.059; p=0.63	r=0.301; p=0.0558	r=0.243; p=0.095	r=-0.047; p=696	r=0.133; p=0.2724	r=0.042; p=0.730	r=-0.210; p=0.084
AR (nuclear)	r=0.212; p=0.088		r=0.124; p=0.306	**r=0.404; p=0.0107**	r=0.2877; p=0.055	r=-0.097; p=0.426	r=0.004; p=0.971	**r=-0.256; p=0.032**	r=0.076; p=0.531
AR (cytoplasmic)	r=-0.059; p=0.63	r=0.124; p=0.306		r=-0.015; p=0.927	r=0.154; p=0.303	r=0.076; p=0.531	r=-0.007; p=0.950	r=-0.085; p=0.483	r=-0.151; p=0.213
FAM111a (nuclear)	r=0.301; p=0.055	**r=0.404; p=0.0107**	r=-0.015; p=0.927		r=0.184; p=0.247	r=0.010; p=0.949	r=-0.071; p=0.656	r=-0.159; p=0.318	r=0. 103; p=0.490
FAM111a (cytoplasmic)	r=0.243; p=0.095	r=0.2877; p=0.055	r=0.154; p=0.309	r=0.184; p=0.247		**r=0.122; p=0.0406**	r=0.317; p=0.830	r=-0.235; p=0.144	r=-0.09; p=0.540
PSA failure	r=-0.047; p=696	r=-0.097; p=0.426	r=0.076; p=0.531	r=0.010; p=0.949	**r=0.122; p=0.0406**		r=0.22; p=0.054	r=0.210; p=0.065	r=0.018; p=0.873
surgical Gleason Grade	r=0.133; p=0.272	r=0.004; p=0.971	r=-0.007; p=0.950	r=-0.071; p=0.656	r=0.317; p=0.830	r=0.22; p=0.054		r=0.078; p=0.493	r=0.057; p=0.618
Pathological stage	r=0.042; p=0.730	**r=-0.256; p=0.032**	r=-0.085; p=0.483	r=-0.085; p=0.483	r=-0.235; p=0.144	r=0.210; p=0.065	r=0.078; p=0.493		r=0.193; p=0.092
Pre-op PSA	r=-0.210; p=0.084	r=0.076; p=0.531	r=-0.151; p=0.213	r=0. 103; p=0.490	r=-0.090; p=0.540	r=0.018; p=0.873	r=0.057; p=0.618	r=0.193; p=0.092	

IF studies of multiple cultured PCa cells defined FAM111A localization in castration-sensitive and resistant PCa cells (Fig. 5C). FAM111A expression in castration-sensitive LNCaP cells is mostly nuclear with intense foci of staining, where a low level of cytoplasmic staining is apparent in some cells. FAM111A localization in complete and steroid depleted media is identical, indicating that localization is not dependent on levels of AR signaling. Likewise, castration-sensitive PC346 cells exhibit almost exclusive nuclear foci staining. In castration-resistant LNCaP derivative C4 cells FAM111A staining persists in nuclear foci but is more dispersed in the cytoplasm. In the progressively more castration-resistant C4-2 cells nuclear foci staining is faint but nuclear and cytoplasmic staining is present. In C4-2 cells we identified a rare mitotic cell which exhibits cytoplasmic FAM111A staining as well as intense staining of mitotic spindle poles. While FAM111A localization to mitotic spindle poles has not been previously observed, it is consistent with FAM111A’s reported role in chromosome segregation and cell cycle regulation [48]. In castration-resistant 22Rv1 cells FAM111A is present in the cytoplasm and nucleus, but focal staining is very weak.

The intense staining of nuclear foci suggests that FAM111A localizes to nucleoli, therefore cells were co-stained with FAM111A and the nucleolar marker NOPP140 (Fig. 5D). In PC346C cells FAM111A and NOPP140 staining overlaps, arguing that in this cellular context FAM111A is highly restricted to the nucleoli. LNCaP exhibit mostly overlapping FAM111A and NOPP140 staining, while in C4-2 cells exhibit some overlap between NOPP140 and FAM111A staining, but FAM111A staining is also dispersed in the nucleus and cytoplasm. In 22Rv1 cells FAM111A is dispersed to other cellular compartments. Notably, perusal of the protein BIOGRID database identified 96 FAM111A interactors, including multiple proteins that localize to the nucleoli.

### FAM111A depletion enhances PARP1 inhibitor efficacy

**Fig. 6:**
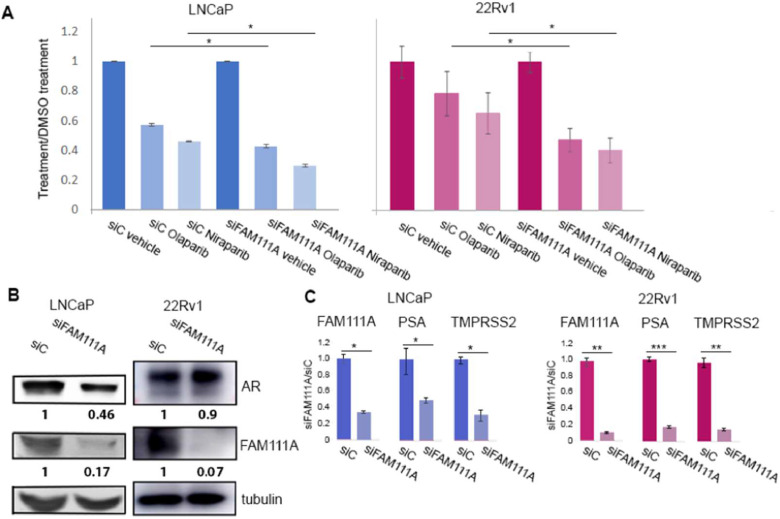
**FAM111A depletion sensitizes cells to PARP inhibitors and reduces AR-dependent transcription. A.** Following transfection with control and FAM111A targeting siRNA cells were treated with olaparib or niraparib (LNCaP-5 uM, 22RV1- 10 and 8uM) for 72 hours. Number of viable cells was adjusted to number of cells following treatment with siC and vehicle which was adjusted to one. **B.** Immunoblots confirm robust FAM111A depletion and AR protein levels. Protein expression was quantified using Image J and standardized to the loading control. **C.** Transcript levels of PSA and TMPRSS2 following FAM111A depletion are significantly reduced. All qRT- PCR analysis was conducted in triplicate using the delta-delta Ct method to calculate fold change in gene expression. * P ≤ 0.05, ** P ≤ 0.005, *** P ≤ 0.0005.

Previous studies using HAP1 chronic myelogenous leukemia cells found that FAM111A depletion sensitizes cells to PARP1 inhibitors [25]. PARP1 inhibitors can trap PARP1 proteins at sites of DNA damage, hindering DNA repair. FAM111A has a role in mitigating such obstacles to facilitate DNA damage repair. Moreover, since PARP1 inhibitors olaparip and niraparib are effective in PCa treatment particularly in patients that have homologous recombination repair alterations, [49,50] we tested whether FAM111A depletion altered the efficacy of the PARP1 inhibitors. LNCaP cells were more sensitive to olaparip and niraparib than 22Rv1 cells but on FAM111A depletion both exhibited greater sensitivity to these PARP1 inhibitors (Fig. 6A), suggesting that cells with reduced FAM111A levels may be more sensitive to these therapeutics.

### FAM111A depletion reduces AR target gene expression

Since AR regulates FAM111A in PCa cells, we asked if there is a feedback or feedforward loop between AR and FAM111A. The analysis was conducted in LNCaP and 22Rv1 cells, where FAM111A localizes primarily to different cellular compartments. Immunoblot analysis confirms siRNA-mediated FAM111A depletion reduces FAM111A protein levels by ∼80 %, while AR protein levels are reduced ∼ 50 % in LNCaP and a modest 10 % in 22Rv1 cells (Fig. 6B). However, transcription of AR-target genes PSA and TMPRSS2 are significantly reduced (60-70 % in LNCaP, ∼80 % in 22Rv1 cells) suggesting that FAM111A depletion hinders AR-dependent transcription, and this activity is not tightly linked to modulation of AR protein levels (Figure 6C).

## Discussion

One well characterized feature of CRPC is the continued reliance on AR signaling, where AR activation circumvents the need for ligand binding [5,51]. Our RNA-seq and subsequent studies revealed that FAM111A, a gene previously identified as harboring PCa predisposing SNPs in two GWAS studies, is AR-regulated in different cellular PCa contexts, suggesting a role for this protein in PCa. We confirmed the binding of AR to a FAM111A site 3′ of the protein coding exons is consistent with AR-mediated repression of this gene. Analysis of 77 different cell types available on ENCODE identified a DNase sensitive region overlapping the AR binding site only in LNCaP cells arguing that AR-mediated FAM111A regulation via this region is restricted to PCa cells.

Overall, in the LNCaP castration resistance progression model and the matched castration-sensitive/resistant LuCaP xenografts FAM111A levels are lower in castration resistant cells. However, in LuCaP136/136CR we observed the opposite effect. One difference between the LuCaP35CR and LuCaP96CR, and LuCaP136CR is that LuCaP136CR tumors exhibited low basal AR signaling evidenced by very low expression of direct AR target genes *PSA, KLK2* and *TMPRSS2*. This is surprising since these cells express abundant amounts of AR protein, higher than in LuCap136 castration sensitive cells and exhibit an ultra sensitivity to abiraterone acetate [36]. Hence, in this cellular context AR is not transactivating known AR target genes despite high AR protein levels, therefore it would be expected that AR-dependent repression would also be divergent from what is encountered in LuCaP35CR and LuCap96CR cells. In cells with low basal levels of AR signaling FAM111A transcription is de-repressed, resulting in elevation of FAM111 transcription.

The IF studies identified a notable change in FAM111A localization between castration sensitive and resistant cells which was particularly apparent in the LNCaP/C4/C42 castration resistance progression model. In parental LNCaP cells FAM111A localization did not change on steroid depletion, hence an acute decrease in AR signaling is not the cause of localization alteration. The castration progression model exhibits two changes- FAM111A transcript levels are decreased with progression and FAM111A localization is progressively redistributed from the nucleoli to the entire nucleus and to the cytoplasm. In castration sensitive PC346 cells FAM111A is restricted to the nucleoli while in castration resistant 22Rv1 cells exhibit weak nucleolar FAM111A staining but staining is distributed in the nucleus and in the cytoplasm, the same distribution observed in C4-2 cells, suggesting that acquisition of castration resistance is co-incident with decreased localization to the nucleoli and greater distribution of FAM111A to the nucleus and cytoplasm. Interestingly, analysis of PCa tumors detected a correlation between FAM111A cytoplasmic staining and PSA failure, buttressing the argument that castration resistance is linked to FAM111A localization alterations. The mechanisms controlling this change are unknown, but it is possible that FAM111A is sequestered in the nucleoli similarly to other proteins that are critical for signal transduction [52] and in castration resistant cells FAM111A is untethered from the nucleoli allowing for release into the nucleus and cytoplasm.

The relocation of FAM111A would likely have effects on nuclear and nuclear membrane proteins. Expression of FAM111A hyperactive mutants is cytotoxic causing disruption of nuclear structure and pore distribution [53]. Nuclear pores serve as gate keepers of molecular transport; thus, changes to these structures would alter trafficking of nuclear and cellular components. In SV40 host range mutant infection, wildtype FAM111A causes nuclear pore, nuclear barrier, and membrane function disruption and an alteration in cell morphology [53]. Proximity labeling of FAM111A and the protease dead FAM111A^S541A^ coupled to mass spectrometry‐based protein analysis identified multiple FAM111A /NUP proteins interactions. Interactions with the protease dead FAM111A were highly enriched when compared to wildtype FAM111A [53]. NUP62 protein levels were lower in cells expressing wildtype FAM111A than the protease dead mutant, suggesting that NUP62 may be a FAM111A target subject to FAM111A-dependent proteolysis. Moreover, cells expressing the cytotoxic, more active FAM111A KCS2 mutant expressed lower levels of NUP62 supporting this notion [53].

In aggressive PCa nuclear pore complex proteins are dysregulated. Nucleoporin POM121 elevation results in increased import of MYC, E2F1, GATA2, and AR into the nucleus, where these oncoproteins promote cell proliferation under castrate conditions [54]. A redistribution of FAM111A into the nuclear compartment could facilitate nuclear translocation of factors that promote proliferation under castrate conditions. Reducing nuclear-cytoplasmic trafficking in conjunction with PARP1 inhibition has therapeutic implications. Inhibition of exportin (XPO1) using selinexor or eltanexor synergizes with PARP1 inhibitors in inducing apoptosis in 22Rv1 cells [55].

Replication fork stalling which occurs on DNA damage promotes double stranded breaks leading to genetic instability. DNA damage response mechanisms critical for maintaining genomic stability and preventing cancer are exploitable vulnerabilities for cancer treatment. PARP1, an enzyme involved in DNA repair to prevent cell death, is crucial for recognizing and responding to DNA breaks, hence a viable drug target [56]. PARP1 inhibitors are particularly effective in cancer cells that have DNA repair deficiencies, especially cells with BRCA1/2 mutations [57]. PARP1 binds to DNA lesions and recruits various DNA repair effectors to the site of the lesion [58], where they are poly-ADP-ribosylated, thus initiating the RNA repair process. Subsequently PARP1 dissociates from the DNA lesions, allowing for efficient DNA damage repair. PARP1 inhibitors interact with PARP1, stabilizing its interaction with DNA leading to PARP1 dysfunction and accumulation of complexes at DNA damage sites [59]. FAM111A has been shown to assist in the resolution of these bulky complexes, an activity that requires FAM111A dimerization and protease activity [26]. Therefore, depletion of FAM111A in PCa cells enhances the cytotoxic effects of PARP1 inhibitors. Since FAM111A levels are lower in PCa metastatic lesions, these cells may be more sensitive to PARP1 inhibitors. However, since there is an increase in FAM111 dispersed in the nucleus rather than confined to the nucleolar compartment in castration resistant cells, more FAM111A may be available to interact with PARP1 to resolve PARP1 complexes, thus hinder PARP1 inhibitor activity. 22Rv1 cells have reduced FAM111A nucleolar expression and are less sensitive to PARP1 inhibitors, but FAM111A depletion in 22Rv1 and LNCaP cells renders cells more sensitive to PARP1 inhibitors indicating that in both cellular contexts FAM111A hinders PARP1 inhibitor efficacy. The observed effect of FAM111A depletion on PARP1 inhibitor-mediated cell viability suggests that tumor cells with very low levels of FAM111A, may be more sensitive to PARP1 inhibitors.

AR signaling is paramount for optimal proliferation and survival of PCa cells. FAM111A elevation following AR depletion may be a mechanism employed to augment expression of AR regulated genes to ensure cell survival when AR signaling is diminished. Depletion of FAM111A in LNCaP had modest effects on AR levels while in 22Rv1 cells AR levels were slightly decreased on FAM111A depletion. However, transcription of PSA and TMPRSS2 was significantly repressed in both cells, therefore, FAM111A-dependent regulation of AR-target gene expression is not tightly linked to AR protein levels. FAM111A may be de-repressed when AR signaling is reduced to allow FAM111A to partly compensate for diminished AR signaling, perhaps to ensure cell survival under suboptimal conditions. How FAM111A is exerting this effect needs to be investigated further, but there are several possible mechanisms leading to reduced AR levels. One possible mechanism is that FAM111A targets proteins such as ubiquitin ligases, kinases, or phosphatases and in the absence of FAM111A an increase of such proteins would lead to reduced AR levels. FAM111A may also affects AR protein levels by altering the integrity of the nuclear membrane and the composition of nuclear proteins. This could lead to changes of AR modifying proteins that affect AR stability. Alternatively, FAM111A may alter nuclear membrane trafficking of transcription factors, thus modifying the expression of multiple genes that encode AR stabilizing proteins such as kinases (CDK1, PIAS1 and STAT5) and molecular chaperones (HSP90, HSP27) [60].

## Conclusion

Castration sensitive and resistant PCa cells are highly reliant on AR signaling. Changes in FAM111A levels and subcellular localization during progression from castration sensitivity to resistance would redistribute this protease into different cellular compartments changing its interactors. Hence AR-dependent regulation of the FAM111A protease gene would alter the proteolysis of protease target proteins thus exerting pleotropic effects on cell physiology. Decreased FAM111A levels or activity would hinder the resolution of replication fork blocking DNA-protein complexes sensitizing cells to PARP1 inhibitors. Additionally, depletion of FAM111A culminates in reduced expression of AR target genes, therefore increased FAM111A expression when AR signaling is limited would serve to augment AR-driven gene expression promoting survival of AR-dependent cells.

Data Availability Statement: All data generated or analyzed during this study are included in this published article (Supplementary data) and available at GEO (GSE286417).

## CRediT authorship contribution statement

**Maria Malvina Tsamouri:** Writing – review & editing, Methodology, Investigation, Funding acquisition, Formal analysis, Data curation. **Stephen J. Libertini:** Methodology, Investigation, Formal analysis, Data curation. **Salma Siddiqui:** Methodology, Investigation, Data curation. **Maitreyee K. Jathal:** Investigation, Funding acquisition, Data curation. **Blythe P. Durbin-Johnson:** Formal analysis. **Clifford G. Tepper:** Writing – review & editing, Investigation, Formal analysis, Data curation. **Eva Corey:** Writing – review & editing, Resources. **Jun Luo:** Resources. **Kenneth A. Iczkowski:** Formal analysis. **Paramita M. Ghosh:** Writing – review & editing, Supervision, Project administration, Funding acquisition, Formal analysis. **Maria Mudryj:** Writing – review & editing, Writing – original draft, Supervision, Resources, Project administration, Funding acquisition, Formal analysis, Conceptualization.

## Declaration of competing interest

The authors declare the following financial interests/personal relationships which may be considered as potential competing interests: EC served as a paid consultant to DotQuant and received Institutional sponsored research funding unrelated to this work from Astra Zeneca, AbbVie, Gilead, Sanofi, Zenith Epigenetics, Bayer Pharmaceuticals, Forma Therapeutics, Genentech, GSK, Janssen Research, Kronos Bio, Foghorn Therapeutics, and MacroGenics. All other authors declare that they have no known competing financial interests or personal relationships that could have appeared to influence the work reported in this paper.

## Appendix Supplementary materials

Image, application 1

Image, application 2
