# Supplementary material for: Alteration in expression and subcellular localization of the androgen receptor- regulated FAM111A protease is associated with emergence of castration resistant prostate cancer
Source: Neoplasia. 2025 May 29;66:101181. doi: 10.1016/j.neo.2025.101181 (PMC12159915; doi:10.1016/j.neo.2025.101181)
Supplement: Supplementary file 1 [file mmc1.pdf]

Alterations in FAM111A subcellular localization and expression during prostate cancer progression.
SUPPLEMENTARY DATA 1

TABLE 1

22Rv1

gene_id	gene name	locus	Rv1- siC (FPKM)	Rv1- siAR (FPKM)	log2(fold_change)	test_stat	p_value	q_value	Fold Change siAR/siC
XLOC_002378	RPS10P7	chr1:201500381-201500877	14.852	0	-1.79769e+308	-1.79769e+308	0.000382839	0.0150375	#VALUE!
C	CSRP1-AS1	chr2:19545654-19545966	31.414	0	-1.79769e+308	1.79769e+308	0.000900522	0.0275722	#VALUE!
XLOC_022366	EGR3	chr8:22545173-22550815	0.2399	5.10821	4.4125	-4.1451	3.40E-05	0.00221768	21.2961392
XLOC_014836	HMOX1	chr22:35777059-35790207	5.0646	103.02	4.3463	-7.83986	4.44E-15	4.86E-12	20.3411593
XLOC_016354	SI	chr3:164696685-164796283	0.8776	17.6687	4.3315	-8.99778	0	0	20.1324382
XLOC_006565	FOS	chr14:75745480-75748937	0.9802	11.3721	3.5363	-5.0655	4.07E-07	6.81E-05	11.6015849
XLOC_023216	RLN2	chr9:5299867-5304580	1.0885	12.3264	3.5014	-3.27891	0.00104208	0.0304862	11.3246142
XLOC_001511	MOB3C	chr1:47073386-47082563	0.7159	7.24883	3.3399	-4.95533	7.22E-07	0.00010824	10.125
XLOC_002419	AKR1C3	chr10:5136567-5149878	0.6762	6.24498	3.2071	-3.10474	0.00190444	0.0485281	9.23479541
XLOC_022993	NIPSNAP3B	chr9:107526450-107536291	0.9624	8.81646	3.1956	-4.54748	5.43E-06	0.00054068	9.16134869
XLOC_022551	GEM	chr8:95261484-95274547	0.9287	8.31764	3.1629	-4.13768	3.51E-05	0.00227154	8.95646853
XLOC_016356	BCHE	chr3:165490691-165555253	1.0022	8.61584	3.1038	-4.95465	7.25E-07	0.00010824	8.59656318
XLOC_021745	PTN	chr7:136912091-137028546	1.4147	12.0146	3.0863	-4.98433	6.22E-07	9.73E-05	8.49297498
XLOC_017135	UGT2B4	chr4:70345882-70361626	0.7649	6.01292	2.9747	-4.06792	4.74E-05	0.00288681	7.86071204
XLOC_010727	TNFSF9	chr19:6531009-6535939	1.5004	11.5122	2.9398	-3.46325	0.000533693	0.0187917	7.67288962
XLOC_023483	ABCA1	chr9:107543283-107690527	0.6835	5.17424	2.9203	-5.82308	5.78E-09	1.58E-06	7.57003515
XLOC_015670	ACPP	chr3:132036210-132087146	4.9728	36.8385	2.8891	-6.77546	1.24E-11	6.43E-09	7.4080303
XLOC_008041	TNFRSF12A	chr16:3070312-3072383	1.3417	9.83725	2.8742	-3.64388	0.000268561	0.0116641	7.33186387
XLOC_021278	AOC1	chr7:150549572-150558379	1.4928	10.9444	2.8741	-5.15703	2.51E-07	4.67E-05	7.33160977
XLOC_004722	NEAT1	chr11:65200487-65201144	3.0637	21.629	2.8196	-3.40618	0.000658795	0.0219428	7.05981315
XLOC_017974	CDH10	chr5:24487208-24645085	0.809	5.67977	2.8116	-4.68582	2.79E-06	0.00030545	7.02043293
XLOC_006320	EFNB2	chr13:107142078-107187388	5.5133	37.3548	2.7603	-6.96498	3.28E-12	2.16E-09	6.77541821
XLOC_005705	MGAT4C	chr12:86373036-87232681	1.2323	7.57633	2.6201	-3.97671	6.99E-05	0.0039366	6.14809731
XLOC_007810	CYP1A1	chr15:75011882-75017877	3.2836	19.0016	2.5328	-4.84795	1.25E-06	0.00016369	5.78681691
XLOC_001786	CTSK	chr1:150768683-150780917	1.1141	5.686	2.3516	-3.70962	0.000207568	0.0095182	5.1038644
XLOC_014400	RBM11	chr21:15588465-15600693	5.8688	29.9312	2.3505	-6.94479	3.79E-12	2.34E-09	5.10004508
XLOC_005161	MYBPC1	chr12:101988746-102079658	1.128	5.70755	2.3391	-4.41906	9.91E-06	0.00084251	5.06000917
XLOC_022994	LOC286367	chr9:107536632-107540045	1.8593	8.7467	2.234	-4.49956	6.81E-06	0.00061974	4.70439761
XLOC_024698	GABRE	chrX:151121595-151143151	1.5965	7.41502	2.2156	-5.12259	3.01E-07	5.35E-05	4.64471476
XLOC_002648	SNCG	chr10:88718287-88723017	2.0695	9.58389	2.2113	-3.09732	0.00195276	0.0493647	4.63105214
XLOC_016210	HGD	chr3:120347014-120401418	2.1128	8.47224	2.0036	-3.62971	0.000283743	0.0121627	4.01004937
XLOC_022212	TRIB1	chr8:126442562-126450644	19.137	76.5895	2.0008	-5.40938	6.32E-08	1.36E-05	4.00227417
XLOC_003602	FAM111A	chr11:58910317-58922511	2.7372	10.9005	1.9936	-5.53608	3.09E-08	7.62E-06	3.98232234

XLOC_002337	-	chr1:149223143-149224125	6.2762	24.9004	1.9882	-3.28877	0.00100625	0.0299629	3.96741689
XLOC_023846	SAT1	chrX:23801274-23804327	4.0368	15.9996	1.9868	-3.48772	0.000487154	0.0177883	3.96345886
XLOC_000467	DNAJB4	chr1:78470635-78482995	2.4596	9.7171	1.9821	-3.64472	0.000267687	0.0116641	3.95070466
XLOC_019466	MAN1A1	chr6:119498400-119670926	7.4305	29.0179	1.9654	-9.29029	0	0	3.90523672
XLOC_004837	GABARAPL1	chr12:10365488-10375724	3.4005	13.1775	1.9542	-3.56738	0.000360565	0.0144505	3.87511735
XLOC_017643	TSLP	chr5:110407389-110413722	1.4321	5.52168	1.947	-3.6497	0.000262544	0.011523	3.85561233
XLOC_015959	CSRNP1	chr3:39183341-39195102	2.7335	10.4449	1.934	-3.54754	0.000388844	0.0151526	3.82107877
XLOC_001615	BCL10	chr1:85731459-85742587	1.939	7.2135	1.8954	-3.73381	0.000188604	0.00885449	3.72019953
XLOC_013417	GCG	chr2:162999384-163008914	19.526	71.0282	1.863	-4.62957	3.66E-06	0.00039698	3.63763301
XLOC_004029	LMNTD2	chr11:537521-560779	3.9774	14.4611	1.8623	-4.04662	5.20E-05	0.00310478	3.63579284
XLOC_018621	HSPA1A	chr6:31783290-31785719	9.3609	33.5081	1.8398	-4.35409	1.34E-05	0.0010798	3.5795792
XLOC_000964	ELF3	chr1:201979689-201986315	5.8448	20.492	1.8098	-4.27575	1.90E-05	0.00140156	3.50598543
XLOC_017727	EGR1	chr5:137801180-137805004	18.686	64.5904	1.7894	-3.94854	7.86E-05	0.00435506	3.45659122
XLOC_017130	UGT2B17	chr4:69402902-69434245	11.48	39.5789	1.7856	-4.36789	1.25E-05	0.00102218	3.44757037
XLOC_021467	IGFBP3	chr7:45951843-45960871	3.1911	10.9751	1.7821	-3.42815	0.0006077	0.0205182	3.43931201
XLOC_006450	LRFN5	chr14:42076763-42373752	3.7363	12.8086	1.7775	-4.03672	5.42E-05	0.00319648	3.42819696
XLOC_005924	SLC16A7	chr12:60177786-60178689	9.4695	32.276	1.7691	-3.61359	0.000301983	0.0127173	3.40841263
XLOC_003798	PLEKHB1	chr11:73357222-73373864	3.7969	12.8401	1.7578	-3.79981	0.000144804	0.00713813	3.38174997
XLOC_002298	-	chr1:16839541-16840397	19.684	62.8437	1.6747	-3.47105	0.000518431	0.0185189	3.19261813
XLOC_022556	TP53INP1	chr8:95938199-95961615	2.6784	8.45019	1.6576	-5.08186	3.74E-07	6.35E-05	3.15497812
XLOC_000905	QSOX1	chr1:180123967-180170147	11.879	36.8747	1.6342	-7.54106	4.66E-14	4.60E-11	3.10417523
XLOC_005590	CALCOCO1	chr12:54104250-54121307	8.5126	26.1403	1.6186	-8.36444	0	0	3.07079031
XLOC_012290	LRATD1	chr2:14772809-14780168	3.9039	11.8705	1.6044	-3.50202	0.000461745	0.0171786	3.04069265
XLOC_021033	PEG10	chr7:94285636-94299006	95.643	289.041	1.5956	-5.26655	1.39E-07	2.80E-05	3.02209707
XLOC_022550	CDH17	chr8:95139393-95229531	9.8045	28.7886	1.554	-4.50331	6.69E-06	0.00061645	2.93626057
XLOC_012282	ATP6V1C2	chr2:10861774-10952960	9.8232	28.5939	1.5414	-4.51843	6.23E-06	0.00058497	2.910849
XLOC_017637	C5orf30	chr5:102594441-102614361	7.2415	21.0292	1.538	-3.84675	0.000119694	0.0060828	2.90397695
XLOC_001664	VAV3	chr1:108113781-108507545	5.748	16.661	1.5354	-4.26516	2.00E-05	0.0014465	2.89858743
XLOC_018622	HSPA1B	chr6:31795511-31798031	15.217	43.9623	1.5306	-3.88982	0.00010032	0.00531752	2.88903964
XLOC_005065	SLC16A7	chr12:60083125-60175408	6.655	18.9616	1.5106	-3.82282	0.000131932	0.00660265	2.84922588
XLOC_000563	GSTM2	chr1:110210643-110226619	8.4449	23.8129	1.4956	-3.30399	0.000953176	0.0288263	2.81981399
XLOC_014551	ADAMTS1	chr21:28208605-28217728	2.7079	7.58611	1.4862	-3.4142	0.000639704	0.0214519	2.80144273
XLOC_009966	PRAC1	chr17:46799081-46799882	52.012	142.601	1.4551	-3.46655	0.000527184	0.0186961	2.74169863
XLOC_002304	-	chr1:24865135-24867479	6.8964	18.7969	1.4466	-3.32331	0.000889573	0.0275722	2.72563051
XLOC_001644	GCLM	chr1:94352589-94375012	6.1193	16.5059	1.4316	-3.15994	0.00157801	0.0415454	2.69738228
XLOC_022640	NDRG1	chr8:134249413-134309547	3.3673	9.05513	1.4272	-3.10093	0.00192914	0.0488931	2.68916821
XLOC_022599	TRPS1	chr8:116420723-116681228	8.4355	22.6577	1.4255	-3.73167	0.000190218	0.00888794	2.68598268
XLOC_023285	AQP3	chr9:33441159-33447590	6.8633	18.3345	1.4176	-3.28659	0.00101407	0.0300232	2.67138887
XLOC_003864	AMOTL1	chr11:94501507-94609918	2.365	6.27165	1.407	-3.44479	0.000571513	0.0195644	2.65188828
XLOC_006429	COCH	chr14:31343740-31359822	7.5928	20.0991	1.4044	-3.39907	0.000676163	0.0224454	2.6471501
XLOC_022157	MATN2	chr8:98881310-99048946	3.5486	9.36836	1.4006	-5.05112	4.39E-07	7.22E-05	2.64004039
XLOC_015922	SATB1	chr3:18389132-18480265	2.2561	5.93919	1.3965	-3.31838	0.000905403	0.0276358	2.63256655

XLOC_001029	ATF3	chr1:212738696-212794117	19.402	50.1043	1.3687	-8.23342	2.22E-16	3.13E-13	2.58243135
XLOC_014410	NCAM2	chr21:22370632-22912517	37.719	96.7852	1.3595	-3.56613	0.000362286	0.0144606	2.56592677
XLOC_005684	CSNK1G1	chr12:72647286-73059422	2.9904	7.66465	1.3579	-3.32121	0.000896295	0.0275722	2.56310041
XLOC_011683	DNAJB1	chr19:14625581-14629201	10.293	26.3325	1.3552	-3.26724	0.00108601	0.031491	2.55839673
XLOC_016877	GAB1	chr4:144257982-144395718	2.2344	5.64804	1.3379	-4.75711	1.96E-06	0.00022779	2.52777841
XLOC_001777	MTMR11	chr1:149900542-149908791	11.885	30.0169	1.3366	-6.92628	4.32E-12	2.51E-09	2.52558921
XLOC_005040	RBMS2	chr12:56915608-57030163	2.1101	5.08171	1.268	-3.60017	0.000318014	0.0132852	2.40824138
XLOC_017131	UGT2B15	chr4:69512314-69536445	50.661	121.863	1.2663	-5.74708	9.08E-09	2.30E-06	2.40545532
XLOC_000183	RCAN3	chr1:24829386-24863843	11.65	27.7213	1.2506	-3.78794	0.000151904	0.00737742	2.37942008
XLOC_016796	PDLIM5	chr4:95373037-95589377	4.6252	10.7913	1.2223	-3.35303	0.00079932	0.025421	2.33313534
XLOC_018154	SEMA6A	chr5:115779250-115910551	35.09	81.5739	1.2171	-3.12225	0.00179475	0.0464422	2.3247249
XLOC_002145	IRF6	chr1:209958967-209979520	3.6705	8.5241	1.2156	-3.64062	0.000271983	0.0117609	2.32232518
XLOC_015930	THRB	chr3:24158644-24536313	5.6291	12.981	1.2054	-3.8334	0.000126387	0.00638998	2.3060759
XLOC_015829	ST6GAL1	chr3:186648314-186796341	4.8971	11.2285	1.1972	-3.87857	0.000105071	0.00551006	2.29287865
XLOC_014446	IFNGR2	chr21:34775201-34852316	8.8048	20.1758	1.1963	-3.40946	0.000650928	0.0217542	2.29144872
XLOC_024070	ARMCX3	chrX:100878119-100882831	5.3124	12.1157	1.1895	-3.3012	0.000962705	0.0290254	2.28067362
XLOC_013127	NRXN1	chr2:50145642-51259674	9.5703	20.6704	1.1109	-3.18308	0.00145715	0.0392515	2.15986329
XLOC_019218	TAPBP	chr6:33267471-33282164	12.509	26.8024	1.0994	-4.16367	3.13E-05	0.00211476	2.14270019
XLOC_004151	SCUBE2	chr11:9041046-9113150	3.4739	7.41969	1.0948	-3.28897	0.00100555	0.0299629	2.13584951
XLOC_006390	ABHD4	chr14:23067146-23081265	8.0179	16.9608	1.0809	-4.30156	1.70E-05	0.00129745	2.11538462
XLOC_001797	SELENBP1	chr1:151336776-151345164	87.355	177.935	1.0264	-6.09074	1.12E-09	3.46E-07	2.03692096
XLOC_018682	CDKN1A	chr6:36644236-36655116	18.491	37.1098	1.005	-4.01546	5.93E-05	0.00340072	2.00695741
XLOC_017284	ANKRD50	chr4:125585203-125633887	6.1939	12.326	0.9928	-4.26215	2.02E-05	0.0014465	1.99001872
XLOC_023540	STOM	chr9:124101352-124132545	19.015	37.619	0.9843	-5.45977	4.77E-08	1.10E-05	1.97833946
XLOC_004222	CD59	chr11:33724555-33758025	3.4003	6.71678	0.9821	-4.18099	2.90E-05	0.00199066	1.97535644
XLOC_021723	UBE2H	chr7:129470572-129592800	26.524	52.2426	0.9779	-4.15648	3.23E-05	0.0021385	1.96966326
XLOC_005586	MAP3K12	chr12:53845885-53893444	10.013	19.3921	0.9537	-3.27897	0.00104187	0.0304862	1.93677049
XLOC_003037	JMJD1C	chr10:64926987-65226322	7.2955	14.1173	0.9524	-3.84841	0.000118887	0.00607307	1.93508508
XLOC_014883	TNRC6B	chr22:40440820-40731812	5.944	11.3707	0.9358	-4.10704	4.01E-05	0.00248497	1.91296702
XLOC_012787	MYO1B	chr2:192110106-192290115	6.6326	12.5249	0.9171	-3.7627	0.000168089	0.00804459	1.88837986
XLOC_017074	SMIM14	chr4:39551602-39640481	20.499	38.5	0.9093	-4.2626	2.02E-05	0.0014465	1.87817826
XLOC_013873	CBFA2T2	chr20:32077927-32237837	2.5303	4.73311	0.9035	-3.27204	0.00106775	0.0310528	1.87060118
XLOC_002137	C1orf116	chr1:207191865-207206101	34.87	64.4575	0.8864	-3.26224	0.00110536	0.0317718	1.84850507
XLOC_000743	EFNA1	chr1:155100348-155107421	13.728	25.2955	0.8818	-3.37307	0.000743353	0.0241076	1.84267163
XLOC_018208	FAM13B	chr5:137225124-137368802	5.5079	10.0519	0.8679	-3.91334	9.10E-05	0.00487751	1.82498929
XLOC_010325	ANKRD20A5P	chr18:14179095-14227049	21.974	39.6774	0.8525	-4.29617	1.74E-05	0.00130782	1.80562588
XLOC_021497	ERV3-1,ZNF117	chr7:64434829-64467124	11.671	20.9567	0.8445	-4.29963	1.71E-05	0.00129745	1.79561991
XLOC_012841	MAP2	chr2:210288770-210598834	3.8448	6.79816	0.8222	-3.20938	0.00133022	0.0364485	1.76815653
XLOC_022071	SDCBP	chr8:59465727-59495419	15.555	27.3881	0.8161	-3.10301	0.00191565	0.0486762	1.76067667
XLOC_016639	PCDH7	chr4:30722029-31148423	9.0985	15.5182	0.7703	-4.46824	7.89E-06	0.00070049	1.70558068
XLOC_022493	NCOA2	chr8:71022850-71316020	16.152	27.4814	0.7667	-5.03269	4.84E-07	7.69E-05	1.70138778
XLOC_013971	NCOA3	chr20:46130600-46285621	10.024	17.0004	0.7621	-3.17473	0.00149974	0.0398543	1.69596101

XLOC_000899	SOAT1	chr1:179262900-179325986	27.018	44.9484	0.7343	-4.10738	4.00E-05	0.00248497	1.66362123
XLOC_004979	PFDN5	chr12:53689234-53693234	96.408	157.721	0.7101	-3.25345	0.00114014	0.0325815	1.63596967
XLOC_015567	NXPE3	chr3:101498028-101579869	21.454	34.5401	0.687	-4.43894	9.04E-06	0.00078873	1.60998249
XLOC_014549	APP	chr21:27252860-27543446	124.3	189.038	0.6048	-6.20619	5.43E-10	1.85E-07	1.52078689
XLOC_005034	MYL6	chr12:56552044-56555366	147.46	220.38	0.5797	-3.50868	0.000450343	0.0168179	1.49451668
XLOC_017181	SEC31A	chr4:83739813-83812419	26.558	37.5616	0.5001	-3.3216	0.000895025	0.0275722	1.41431943
XLOC_009077	WSB1	chr17:25621105-25640658	32.502	45.1265	0.4734	-3.28173	0.00103174	0.0304549	1.38841995
XLOC_005857	RSRC2	chr12:122989189-123011560	45.333	62.4431	0.462	-3.21716	0.00129468	0.0358546	1.37742809
XLOC_002231	ARID4B	chr1:235330209-235491532	15.205	20.6475	0.4414	-3.51877	0.000433557	0.0164317	1.3579346
XLOC_015787	FXR1	chr3:180630233-180700539	20.397	27.5081	0.4315	-5.17577	2.27E-07	4.30E-05	1.3486257
XLOC_006989	NUMB	chr14:73741917-73925286	16.831	22.0507	0.3897	-3.47607	0.00050882	0.0183082	1.3101046
XLOC_011032	SPINT2	chr19:38755097-38783254	156.79	205	0.3867	-3.69276	0.000221836	0.00985173	1.30744295
XLOC_009242	NBR1	chr17:41322497-41363707	22.856	29.2544	0.3561	-3.92897	8.53E-05	0.0046727	1.27996734
XLOC_020974	GTF2I	chr7:74072029-74175022	60.092	73.0108	0.2809	-3.26005	0.00111392	0.0319249	1.21497616
XLOC_004984	PCBP2	chr12:53845885-53893444	64.969	75.478	0.2163	-4.87009	1.12E-06	0.00015274	1.16175351
XLOC_004998	HNRNPA1	chr12:54674487-54679358	119.56	100.778	-0.2466	3.26503	0.00109453	0.0316451	0.84288692
XLOC_017860	NPM1	chr5:170814707-170837888	415.19	324.594	-0.3551	5.87932	4.12E-09	1.19E-06	0.78180028
XLOC_017910	CANX	chr5:179125929-179158639	388.49	292.289	-0.4105	3.80825	0.000139955	0.00693375	0.75236936
XLOC_004523	SYTL2	chr11:85405264-85522178	45.583	33.4556	-0.4463	3.45465	0.000551015	0.019264	0.73394405
XLOC_010784	ILF3	chr19:10764936-10803095	58.657	42.9638	-0.4492	3.72665	0.000194039	0.00902374	0.73245498
XLOC_004909	DNM1L	chr12:32832136-32898584	26.068	18.5116	-0.4939	3.49809	0.000468604	0.0173032	0.71011963
XLOC_018184	VDAC1	chr5:133307565-133340824	67.394	47.7784	-0.4963	3.0965	0.00195818	0.0493752	0.7089388
XLOC_010793	LDLR	chr19:11200037-11244505	26.435	18.2792	-0.5323	3.11016	0.00186988	0.0481335	0.69146494
XLOC_022468	ASPH	chr8:62200524-62627199	53.599	36.6997	-0.5464	6.08026	1.20E-09	3.58E-07	0.68470384
XLOC_002240	HEATR1	chr1:236681513-236767841	27.179	18.5443	-0.5515	4.48196	7.40E-06	0.00066288	0.68231366
XLOC_005471	SINHCAF	chr12:31433519-31479159	31.852	21.6434	-0.5575	3.34067	0.000835752	0.026241	0.67949602
XLOC_013240	IMMT	chr2:86371054-86422893	20.994	14.2458	-0.5595	3.48627	0.000489815	0.017816	0.67855281
XLOC_013950	PIGT	chr20:44044706-44054884	41.166	27.9035	-0.561	3.21065	0.00132436	0.0364485	0.67783874
XLOC_011654	TNPO2	chr19:12810007-12834810	20.125	13.6245	-0.5628	4.17556	2.97E-05	0.00202108	0.67699449
XLOC_017799	ARHGEF37	chr5:148961134-149014529	30.109	20.373	-0.5635	3.20244	0.00136269	0.0372153	0.67664921
XLOC_006913	NIN	chr14:51186480-51297839	22.469	15.1908	-0.5647	3.57764	0.000346706	0.0140089	0.67608568
XLOC_015345	MLH1	chr3:37034840-37092337	39.913	26.4869	-0.5916	3.24152	0.00118892	0.0334902	0.66361344
XLOC_012503	MAT2A	chr2:85766100-85788657	133.25	87.7599	-0.6025	4.18059	2.91E-05	0.00199066	0.65862172
XLOC_020917	CCT6A	chr7:56119377-56131682	114.19	75.1282	-0.6041	4.11897	3.81E-05	0.00240511	0.657904
XLOC_005212	ERP29	chr12:112451151-112461024	88.058	57.7694	-0.6082	3.37539	0.000737113	0.0239841	0.65603514
XLOC_005336	SCNN1A	chr12:6456008-6486523	25.094	16.2405	-0.6277	3.25195	0.00114616	0.0326589	0.64719977
XLOC_004356	BSCL2	chr11:62457733-62494857	67.662	43.7266	-0.6298	4.30044	1.70E-05	0.00129745	0.64624808
XLOC_024424	STK4	chrX:53962634-54071569	30.878	19.5908	-0.6564	4.20508	2.61E-05	0.0018379	0.63446391
XLOC_000364	NASP	chr1:46049659-46084578	54.351	34.2627	-0.6657	3.57781	0.000346479	0.0140089	0.6303985
XLOC_021593	ASNS	chr7:97481428-97501854	332.84	209.552	-0.6675	3.47324	0.000514224	0.0184354	0.62959239
XLOC_005047	SHMT2	chr12:57623355-57634475	121.52	76.3275	-0.6709	8.12535	4.44E-16	5.47E-13	0.62811386
XLOC_009491	ANAPC11	chr17:79849598-79858363	63.364	39.7974	-0.671	3.52837	0.000418134	0.0159782	0.62807206

XLOC_005545	SLC11A2	chr12:51373565-51422058	82.703	51.9352	-0.6712	6.42688	1.30E-10	5.35E-08	0.62797542
XLOC_008965	EIF5A	chr17:7210317-7215782	96.858	60.6247	-0.676	3.1251	0.00177745	0.0461154	0.62591515
XLOC_024381	SSX3	chrX:48205809-48216142	96.601	60.1219	-0.6841	4.21521	2.50E-05	0.00176996	0.62237542
XLOC_015478	GNL3	chr3:52719935-52742197	61.64	37.8382	-0.704	4.69866	2.62E-06	0.00029009	0.61386401
XLOC_019297	GTPBP2	chr6:43543877-43596936	42.402	25.9804	-0.7067	3.85446	0.000115984	0.00599603	0.61271624
XLOC_007079	WARS	chr14:100800124-100842680	30.817	18.7888	-0.7138	4.02124	5.79E-05	0.00333782	0.60969389
XLOC_000230	RCC1	chr1:28832454-28865708	127.77	77.2085	-0.7267	3.55836	0.000373184	0.0147543	0.60429211
XLOC_021973	SLC39A14	chr8:22224761-22291640	44.409	26.7891	-0.7292	3.7377	0.000185715	0.00876059	0.60323164
XLOC_018237	DIAPH1	chr5:140894587-140998622	15.609	9.35056	-0.7393	3.38845	0.000702896	0.0230995	0.59903367
XLOC_001868	GBA	chr1:155204193-155214653	38.06	22.7832	-0.7403	4.13227	3.59E-05	0.00228609	0.59861777
XLOC_000740	ADAM15	chr1:155006281-155042029	17.027	10.1688	-0.7436	3.44667	0.000567539	0.0195644	0.59723599
XLOC_006449	MIA2	chr14:39734475-39820397	40.783	24.1591	-0.7554	6.46786	9.94E-11	4.26E-08	0.59238951
XLOC_013943	PABPC1L	chr20:43538702-43568971	52.025	30.497	-0.7705	3.58399	0.000338381	0.0138158	0.5861968
XLOC_008111	CCP110	chr16:19535178-19564728	7.9538	4.59095	-0.7928	3.32582	0.000881578	0.0274179	0.57720512
XLOC_001669	CLCC1	chr1:109419602-109506111	24.108	13.7568	-0.8094	4.80486	1.55E-06	0.00018849	0.57063415
XLOC_004689	SRPR	chr11:126081618-126138877	63.328	35.9755	-0.8158	7.33159	2.27E-13	1.88E-10	0.56808236
XLOC_021179	MEST	chr7:130126045-130371406	70.94	40.2165	-0.8188	4.56326	5.04E-06	0.00051069	0.56691054
XLOC_011124	PVR	chr19:45147097-45169428	22.023	12.4323	-0.8249	3.3762	0.000734934	0.0239841	0.5645182
XLOC_003664	SLC3A2	chr11:62623483-62656358	147.74	83.1108	-0.8299	3.15875	0.00158445	0.0415454	0.56255108
XLOC_017846	WWC1	chr5:167719064-167899308	11.632	6.49516	-0.8406	5.52614	3.27E-08	7.87E-06	0.55839309
XLOC_023445	PTCH1	chr9:98205263-98279247	12.541	6.97	-0.8475	4.84567	1.26E-06	0.00016369	0.55575695
XLOC_017493	SKP2	chr5:36152144-36184142	25.183	13.939	-0.8533	3.22727	0.00124976	0.0347082	0.55350567
XLOC_015867	LMLN	chr3:197687070-197770591	18.178	10.0589	-0.8537	3.55769	0.000374133	0.0147543	0.55335913
XLOC_008296	CKLF	chr16:66586465-66613038	45.218	24.8964	-0.861	3.10467	0.0019049	0.0485281	0.55058912
XLOC_016270	TOPBP1	chr3:133319448-133380737	20.818	11.4563	-0.8617	3.42936	0.000605016	0.0204993	0.55029648
XLOC_021456	H2AFV	chr7:44866487-44887725	64.165	35.0867	-0.8709	3.29526	0.000983308	0.0295562	0.54682231
XLOC_018620	MSH5	chr6:31707724-31732806	37.678	20.5538	-0.8743	3.27771	0.00104653	0.0305258	0.54550904
XLOC_005496	SLC38A1	chr12:46576840-46663208	90.661	49.3715	-0.8768	5.12117	3.04E-07	5.35E-05	0.54457324
XLOC_007687	CEP152	chr15:49030134-49103343	19.483	10.5831	-0.8805	4.75313	2.00E-06	0.00022961	0.54318931
XLOC_005147	TMPO	chr12:98906750-98944157	20.355	11.0515	-0.8811	3.22749	0.0012488	0.0347082	0.54294501
XLOC_002064	UCHL5	chr1:192981495-193060906	71.583	38.7866	-0.8841	4.59968	4.23E-06	0.00045288	0.54183895
XLOC_020919	SUMF2	chr7:56131916-56148367	74.377	40.1321	-0.8901	5.41457	6.14E-08	1.35E-05	0.53957372
XLOC_014601	GART	chr21:34876237-34915198	39.689	21.1725	-0.9066	3.79366	0.000148444	0.00728116	0.53345593
XLOC_020931	ZNF107	chr7:64126510-64171401	15.195	7.99957	-0.9256	3.18672	0.00143895	0.0389742	0.52646736
XLOC_022988	SMC2	chr9:106856540-106903700	22.565	11.8474	-0.9295	4.02289	5.75E-05	0.00333392	0.52502827
XLOC_009067	ALDH3A2	chr17:19552063-19580908	266.03	139.285	-0.9336	5.03799	4.70E-07	7.60E-05	0.52356189
XLOC_004902	DDX11	chr12:31173696-31257725	11.006	5.7482	-0.9371	3.28052	0.00103617	0.0304862	0.52228892
XLOC_012358	SLC30A6	chr2:32390909-32449181	13.474	6.99772	-0.9452	3.445	0.000571057	0.0195644	0.51935899
XLOC_001466	PPT1	chr1:40538381-40563142	31.154	16.0256	-0.959	4.52463	6.05E-06	0.0005791	0.51439624
XLOC_004425	SLC29A2	chr11:66129991-66139291	32.783	16.7172	-0.9716	4.45228	8.50E-06	0.00074789	0.50993349
XLOC_011609	DNMT1	chr19:10244021-10305755	19.463	9.78588	-0.992	4.8221	1.42E-06	0.00017728	0.50278273
XLOC_005726	CEP83	chr12:94702055-94853764	19.27	9.67479	-0.994	3.56933	0.000357895	0.014402	0.50207612

XLOC_017996	NUP155	chr5:37291940-37371197	27.093	13.6019	-0.9941	4.59769	4.27E-06	0.00045288	0.5020441
XLOC_005984	GTF3A	chr13:27998680-28024739	38.768	19.4391	-0.9959	3.15425	0.00160913	0.0420808	0.50142541
XLOC_014121	TMX4	chr20:7960695-8000393	28.93	14.4633	-1.0002	3.79093	0.000150084	0.00732515	0.49993416
XLOC_016224	HACD2	chr3:123212185-123303924	57.453	28.5546	-1.0087	4.31403	1.60E-05	0.00124446	0.49701111
XLOC_004960	SLC4A8	chr12:51818593-51902980	11.147	5.52264	-1.0132	3.69637	0.000218704	0.00980091	0.49544266
XLOC_005865	MPHOSPH9	chr12:123640012-123706445	28.526	14.1109	-1.0155	5.78254	7.36E-09	1.91E-06	0.49466715
XLOC_012440	SLC1A4	chr2:65215578-65251000	12.231	6.02697	-1.021	3.70001	0.000215589	0.00970544	0.4927815
XLOC_017360	HMGB2	chr4:174252526-174255595	112.99	54.8248	-1.0433	3.68486	0.000228828	0.0101166	0.4852096
XLOC_000494	LRRC8D	chr1:90286572-90401989	21.901	10.604	-1.0464	3.3677	0.000757971	0.0245011	0.48418826
XLOC_016451	TFRC	chr3:195776154-195809032	56.673	27.0576	-1.0666	3.18564	0.00144434	0.0390129	0.47743625
XLOC_013786	MCM8	chr20:5931297-5976303	21.604	10.234	-1.0779	4.90159	9.51E-07	0.00013783	0.47370802
XLOC_001355	STMN1	chr1:26210676-26233368	34.918	16.5371	-1.0783	3.89697	9.74E-05	0.00519073	0.47359311
XLOC_009447	LOC100507246	chr17:74553845-74582145	43.672	20.4266	-1.0963	6.10569	1.02E-09	3.26E-07	0.46772745
XLOC_021613	MCM7	chr7:99690304-99723128	40.742	18.9677	-1.103	3.98827	6.66E-05	0.00377317	0.46555709
XLOC_023992	AR	chrX:66763873-66946301	131.66	61.29	-1.1031	3.91837	8.91E-05	0.0048028	0.46551514
XLOC_016397	ALG3	chr3:183948316-183967313	46.374	21.5131	-1.1081	3.81227	0.000137698	0.00685637	0.46390137
XLOC_018735	SLC29A1	chr6:44187241-44201918	29.798	13.7697	-1.1137	4.73606	2.18E-06	0.00024694	0.46210417
XLOC_018854	AMD1	chr6:111195986-111217643	53.745	24.7411	-1.1192	4.04388	5.26E-05	0.00312249	0.46034903
XLOC_005230	RFC5	chr12:118454505-118498951	13.243	6.08437	-1.122	3.41554	0.000636559	0.0214192	0.45945644
XLOC_022907	CEP78	chr9:80850990-80881983	103.25	47.3283	-1.1254	6.37756	1.80E-10	6.94E-08	0.4583908
XLOC_009251	G6PC3	chr17:42148097-42153712	65.965	30.1895	-1.1276	3.61794	0.000296963	0.0125655	0.45766377
XLOC_006057	CKAP2	chr13:53029494-53050763	34.807	15.8217	-1.1375	5.23397	1.66E-07	3.27E-05	0.45455287
XLOC_002215	TTC13	chr1:231041986-231114618	14.745	6.67817	-1.1427	3.44734	0.000566131	0.0195644	0.45290488
XLOC_005262	KNTC1	chr12:123011808-123110947	35.715	16.0495	-1.154	6.1374	8.39E-10	2.76E-07	0.44938379
XLOC_015754	SMC4	chr3:160117429-160152778	17.76	7.91701	-1.1656	5.40263	6.57E-08	1.36E-05	0.4457881
XLOC_008692	NETO2	chr16:47115430-47177936	23.623	10.3548	-1.1899	3.85409	0.000116162	0.00599603	0.43833628
XLOC_003424	WEE1	chr11:9595227-9611313	22.915	10.0424	-1.1902	3.87115	0.000108321	0.00565047	0.43825425
XLOC_021051	ZNF789	chr7:99070514-99085217	117.54	51.4808	-1.1911	4.53665	5.72E-06	0.00055792	0.43797183
XLOC_013805	MGME1	chr20:17949744-17971762	22.486	9.81194	-1.1964	3.30922	0.000935575	0.028381	0.43635372
XLOC_000213	TRNP1	chr1:27320194-27328101	41.264	17.9895	-1.1977	5.22727	1.72E-07	3.33E-05	0.43596372
XLOC_003999	FOXRED1	chr11:126138934-126148027	14.894	6.48895	-1.1987	3.96453	7.35E-05	0.00409619	0.43567372
XLOC_012201	ZBTB45	chr19:59024896-59030921	19.105	8.29703	-1.2033	3.34452	0.000824261	0.0259629	0.43429282
XLOC_000861	C1orf112	chr1:169764199-169863076	11.711	5.06175	-1.2102	3.50004	0.000465182	0.0172414	0.43220869
XLOC_007902	PRC1	chr15:91509267-91537804	33.421	14.4328	-1.2114	6.66748	2.60E-11	1.28E-08	0.43184336
XLOC_010284	TYMS	chr18:596997-712662	51.869	22.3912	-1.2119	5.14725	2.64E-07	4.83E-05	0.43169072
XLOC_009314	EME1	chr17:48450580-48474914	22.41	9.67049	-1.2125	4.19358	2.75E-05	0.00191995	0.43153516
XLOC_000844	UCK2	chr1:165796731-165880855	18.478	7.97258	-1.2127	3.16762	0.00153693	0.0407327	0.43146038
XLOC_017677	LMNB1	chr5:126112314-126172712	25.701	11.0819	-1.2136	3.42934	0.000605061	0.0204993	0.43118533
XLOC_001541	LRP8	chr1:53692563-53793821	7.9834	3.4401	-1.2146	4.79341	1.64E-06	0.00019715	0.43090448
XLOC_017805	TCOF1	chr5:149737201-149779871	6.8051	2.91978	-1.2207	3.17989	0.00147332	0.039264	0.42906258
XLOC_005326	FOXM1	chr12:2945981-2986321	14.443	6.18775	-1.2229	4.53068	5.88E-06	0.00056828	0.42842661
XLOC_002186	LBR	chr1:225589203-225616557	27.933	11.9233	-1.2282	3.19988	0.00137483	0.0374433	0.42684672

XLOC_008117	REXO5	chr16:20775311-20860990	13.603	5.80398	-1.2288	4.15343	3.28E-05	0.0021527	0.42666036
XLOC_000618	PHGDH	chr1:120254418-120286849	55.314	23.5588	-1.2314	3.24549	0.00117248	0.0332168	0.4259069
XLOC_001119	EXO1	chr1:242011492-242053241	12.268	5.22217	-1.2321	4.53814	5.68E-06	0.00055792	0.42568849
XLOC_011580	CD320	chr19:8367010-8373240	25.714	10.9378	-1.2332	3.69473	0.00022012	0.00981974	0.42536405
XLOC_000935	RGS2	chr1:192778168-192781407	175.3	74.3947	-1.2365	3.24393	0.00117892	0.0333036	0.42438924
XLOC_004937	TROAP	chr12:49716970-49725516	18.025	7.60776	-1.2444	3.24715	0.00116566	0.0331189	0.42207755
XLOC_016465	BDH1	chr3:197236653-197300194	7.6621	3.22318	-1.2493	3.36214	0.000773407	0.0247566	0.42066392
XLOC_020912	EGFR	chr7:55086695-55279369	113.79	47.6161	-1.2568	7.3125	2.62E-13	1.96E-10	0.41847693
XLOC_007283	KNL1	chr15:40886446-40954881	6.2928	2.62105	-1.2636	4.2694	1.96E-05	0.0014314	0.41651778
XLOC_007275	BUB1B	chr15:40453209-40569688	19.058	7.91226	-1.2683	4.56161	5.08E-06	0.00051069	0.41516019
XLOC_016756	FRAS1	chr4:78978723-79465423	7.608	3.15078	-1.2718	3.23239	0.0012276	0.0343832	0.41413987
XLOC_009717	SPAG5	chr17:26904582-26926056	15.134	6.2529	-1.2752	3.1806	0.00146969	0.039264	0.4131822
XLOC_004175	CALCA	chr11:14988214-14993870	43.354	17.9032	-1.276	4.03557	5.45E-05	0.00319648	0.41295028
XLOC_015292	TSEN2	chr3:12525930-12574820	14.052	5.77792	-1.2821	3.33044	0.000867083	0.0270524	0.41118798
XLOC_008868	FANCA	chr16:89773540-89883065	14.253	5.85507	-1.2835	3.36231	0.00077294	0.0247566	0.41078916
XLOC_009084	TMEM97	chr17:26646120-26662495	20.96	8.57334	-1.2897	4.28225	1.85E-05	0.00138187	0.40903608
XLOC_000604	ATP1A1	chr1:116915794-116961244	806.23	327.677	-1.2989	5.45028	5.03E-08	1.13E-05	0.40643315
XLOC_017987	PRLR	chr5:35048860-35230823	23.245	9.40714	-1.3051	6.23694	4.46E-10	1.57E-07	0.40469306
XLOC_016348	TRIM59	chr3:160153290-160167626	16.821	6.79725	-1.3073	4.49824	6.85E-06	0.00061974	0.4040876
XLOC_004362	SNHG1	chr11:62619457-62623360	144.81	58.419	-1.3097	4.07755	4.55E-05	0.00278704	0.40341315
XLOC_020804	BZW2	chr7:16685758-16746148	34.756	13.9778	-1.3141	3.38517	0.000711353	0.0232998	0.40216794
XLOC_006928	WDHD1	chr14:55405655-55493819	7.3723	2.9594	-1.3168	3.10716	0.00188892	0.0483712	0.40142434
XLOC_023301	SIGMAR1	chr9:34634718-34637768	190.1	75.8292	-1.326	8.256	2.22E-16	3.13E-13	0.39888368
XLOC_009723	TLCD1	chr17:27046935-27053949	30.234	12.0338	-1.3291	3.59235	0.000327712	0.0135268	0.39802198
XLOC_009784	MYO19	chr17:34842472-34891305	12.983	5.1433	-1.3359	3.48533	0.000491525	0.017816	0.39615859
XLOC_014276	SLC13A3	chr20:45186461-45318276	26.189	10.3504	-1.3393	6.3749	1.83E-10	6.94E-08	0.39521785
XLOC_009298	ATP5G1	chr17:46970147-46973232	109.45	43.2228	-1.3404	3.59113	0.000329245	0.0135268	0.3949221
XLOC_016938	GALNT7	chr4:174089903-174245118	25.997	10.2391	-1.3442	3.43636	0.000589579	0.020113	0.39386415
XLOC_002439	DHTKD1	chr10:12110933-12165224	12.172	4.78724	-1.3463	3.32011	0.000899815	0.0275722	0.39330488
XLOC_016731	ALB	chr4:74269971-74287129	39.591	15.5176	-1.3513	3.35887	0.000782616	0.0249703	0.39194958
XLOC_002125	SLC45A3	chr1:205626980-205649630	48.166	18.8255	-1.3553	3.53969	0.000400595	0.0154881	0.39084541
XLOC_000734	CKS1B	chr1:154947117-154951725	90.783	35.4232	-1.3577	3.58342	0.000339124	0.0138158	0.39019846
XLOC_015403	LIMD1	chr3:45636322-45730374	24.323	9.47966	-1.3594	4.42745	9.54E-06	0.00081746	0.38973894
XLOC_004774	RAD51AP1	chr12:4647949-4669213	27.998	10.8561	-1.3668	3.36638	0.000761627	0.0245388	0.38774229
XLOC_023099	LRRC8A	chr9:131644390-131680317	120.39	46.4671	-1.3734	3.72451	0.000195699	0.00905818	0.38598856
XLOC_016212	POLQ	chr3:121150272-121264853	6.62	2.54898	-1.3769	3.33817	0.000843314	0.0263944	0.38503994
XLOC_017277	CCNA2	chr4:122722471-122745088	14.846	5.68561	-1.3847	3.10733	0.00188783	0.0483712	0.38297975
XLOC_015926	SGO1	chr3:20202084-20227724	13.795	5.27164	-1.3878	3.84966	0.000118283	0.00607307	0.38215505
XLOC_010338	LAMA3	chr18:21269561-21535029	5.5981	2.1377	-1.3889	4.33954	1.43E-05	0.00114446	0.38186643
XLOC_009071	DHRS7B	chr17:21030257-21094836	31.506	12.0035	-1.3922	3.26288	0.00110288	0.0317718	0.38099659
XLOC_015397	KIF15	chr3:44803208-44894748	15.31	5.82153	-1.395	3.52503	0.000423428	0.016118	0.38023415
XLOC_001022	TRAF5	chr1:211499956-211548286	8.6009	3.2642	-1.3978	4.13213	3.59E-05	0.00228609	0.37952057

XLOC_012279	RRM2	chr2:10262694-10271546	53.92	20.4283	-1.4002	3.59684	0.0003221	0.0133991	0.37886611
XLOC_019328	MCM3	chr6:52128811-52149582	18.909	7.15773	-1.4015	3.34916	0.000810558	0.0256955	0.37853012
XLOC_001249	SRM	chr1:11114648-11120091	58.952	21.9974	-1.4222	3.44517	0.0005707	0.0195644	0.37314028
XLOC_019239	FKBP5	chr6:35541297-35704724	62.989	23.5005	-1.4224	3.88552	0.000102109	0.00538338	0.37308855
XLOC_004339	FADS1	chr11:61567096-61584529	111.34	41.1443	-1.4362	3.61261	0.00030313	0.0127173	0.36953425
XLOC_013956	UBE2C	chr20:44441254-44445596	46.502	17.1314	-1.4406	4.88298	1.04E-06	0.0001451	0.36840384
XLOC_000609	TTF2	chr1:117602948-117645491	7.4799	2.75389	-1.4416	3.23975	0.00119636	0.0336038	0.36817154
XLOC_001518	STIL	chr1:47715810-47779819	7.3709	2.70827	-1.4445	3.75689	0.000172036	0.00819376	0.36742711
XLOC_023886	MAOA	chrX:43515408-43606068	27.072	9.85336	-1.4581	3.48807	0.000486529	0.0177883	0.36396963
XLOC_006409	PCK2	chr14:24563482-24573339	145.37	52.7686	-1.4619	3.61868	0.000296108	0.0125655	0.36300467
XLOC_022214	MYC	chr8:128748314-128753680	50.379	18.2747	-1.463	3.82786	0.000129263	0.00650205	0.36274559
XLOC_007294	NUSAP1	chr15:41624891-41673248	36.427	13.2115	-1.4632	8.17072	2.22E-16	3.13E-13	0.36268023
XLOC_005636	NEMP1	chr12:57449425-57472574	48.978	17.7	-1.4684	10.2357	0	0	0.36138286
XLOC_009198	CDC6	chr17:38444145-38459413	19.525	7.03396	-1.4729	3.4522	0.000556033	0.0193708	0.36025241
XLOC_005157	GAS2L3	chr12:100967488-101018685	31.022	11.0381	-1.4908	3.71121	0.00020627	0.00950288	0.35581765
XLOC_005828	CIT	chr12:120123594-120315095	7.9396	2.81452	-1.4962	3.93499	8.32E-05	0.00458257	0.35449078
XLOC_022000	ESCO2	chr8:27632057-27662424	11.446	4.02031	-1.5095	3.31162	0.00092759	0.0282257	0.35123536
XLOC_001544	NDC1	chr1:54231133-54304225	31.524	11.0711	-1.5096	4.84232	1.28E-06	0.00016432	0.35119884
XLOC_009463	BIRC5	chr17:76210276-76221716	23.418	8.20307	-1.5134	5.4579	4.82E-08	1.10E-05	0.35028473
XLOC_002150	NEK2	chr1:211831598-211848972	36.86	12.9101	-1.5136	4.81821	1.45E-06	0.00017851	0.35024588
XLOC_007410	KIF23	chr15:69706626-69740764	23.229	8.11955	-1.5165	6.50407	7.82E-11	3.50E-08	0.34954013
XLOC_021420	NT5C3	chr7:33053741-33102409	17.923	6.22936	-1.5247	4.16091	3.17E-05	0.00212596	0.34756146
XLOC_013851	TPX2	chr20:30326903-30389603	51.628	17.9361	-1.5253	3.98814	6.66E-05	0.00377317	0.34741212
XLOC_006929	DLGAP5	chr14:55614833-55658396	14.047	4.87346	-1.5272	4.02346	5.73E-05	0.00333392	0.34695008
XLOC_021791	EZH2	chr7:148504342-148581441	44.286	15.3178	-1.5316	7.33051	2.29E-13	1.88E-10	0.34588635
XLOC_015076	PLA2G3	chr22:31530792-31536469	15.566	5.35425	-1.5396	3.49088	0.000481433	0.0177106	0.34398081
XLOC_007779	CLN6	chr15:68499329-68522080	18.914	6.445	-1.5532	3.4652	0.000529843	0.018723	0.34075813
XLOC_021992	CDCA2	chr8:25316512-25365425	14.138	4.80009	-1.5585	3.96946	7.20E-05	0.00403518	0.3395133
XLOC_009926	KIF18B	chr17:43003258-43025082	8.8453	2.9969	-1.5614	3.51788	0.000435002	0.0164317	0.33881273
XLOC_014715	CDC45	chr22:19467413-19508135	13.361	4.49683	-1.571	3.51562	0.000438733	0.0165094	0.33657735
XLOC_003992	CHEK1	chr11:125495035-125550793	7.3378	2.43182	-1.5933	3.4695	0.000521419	0.0185584	0.33141022
XLOC_015775	ECT2	chr3:172472297-172539263	35.808	11.8437	-1.5962	5.40159	6.61E-08	1.36E-05	0.33075388
XLOC_005540	RACGAP1	chr12:50382944-50419307	38.394	12.5994	-1.6075	4.58306	4.58E-06	0.00047554	0.32816425
XLOC_015279	FANCD2	chr3:10068112-10149915	20.063	6.55179	-1.6146	4.15733	3.22E-05	0.0021385	0.32656227
XLOC_013640	HJURP	chr2:234745485-234763212	13.861	4.513	-1.6189	3.5911	0.000329285	0.0135268	0.32559264
XLOC_020777	RBAK	chr7:5085451-5112854	35.972	11.6923	-1.6213	4.18374	2.87E-05	0.00199066	0.32503568
XLOC_003272	MKI67	chr10:129894924-129924468	34.89	11.3268	-1.6231	6.84321	7.74E-12	4.24E-09	0.3246394
XLOC_004211	KIF18A	chr11:28042162-28129746	9.0165	2.90366	-1.6347	3.13632	0.0017108	0.0445034	0.32203737
XLOC_010621	-	chr18:75694390-75696299	23.56	7.57236	-1.6375	3.53449	0.000408561	0.0156732	0.3214085
XLOC_008992	TMEM88	chr17:7758383-7759417	47.442	15.2057	-1.6416	3.63214	0.000281076	0.012101	0.32051194
XLOC_010622	-	chr18:75696833-75699451	43.161	13.8205	-1.6429	4.10383	4.06E-05	0.00250402	0.32020994
XLOC_008822	MAF	chr16:79627744-79634622	4.475	1.42885	-1.647	3.28801	0.001009	0.0299629	0.3192968

XLOC_009901	BRCA1	chr17:41196311-41277500	10.857	3.46528	-1.6476	4.96739	6.79E-07	0.00010454	0.31916846
XLOC_017470	OTULINL	chr5:14581890-14616287	4.4404	1.41493	-1.65	3.16544	0.00154849	0.040929	0.3186512
XLOC_012540	NCAPH	chr2:97001483-97041274	8.2132	2.61089	-1.6534	3.54081	0.0003989	0.0154833	0.3178901
XLOC_002681	KIF11	chr10:94352824-94415152	11.576	3.67031	-1.6572	3.92444	8.69E-05	0.00473515	0.31705829
XLOC_001335	E2F2	chr1:23832919-23857712	4.5144	1.42896	-1.6596	3.21069	0.00132419	0.0364485	0.31653567
XLOC_017836	PTTG1	chr5:159848864-159855746	57.361	18.0887	-1.665	3.70125	0.000214537	0.00970544	0.31534654
XLOC_015335	CMTM7	chr3:32433162-32496333	14.931	4.70254	-1.6668	3.11398	0.00184582	0.0476386	0.31495334
XLOC_005952	-	chr12:125514139-125515730	38.458	12.1065	-1.6675	3.74846	0.000177927	0.00843355	0.31479619
XLOC_013931	MYBL2	chr20:42295708-42345122	8.4016	2.63596	-1.6723	3.20923	0.00133091	0.0364486	0.31374405
XLOC_018887	SMPDL3A	chr6:123109970-123130864	10.649	3.33353	-1.6756	3.17984	0.00147354	0.039264	0.31302939
XLOC_019553	FBXO5	chr6:153291657-153304740	9.8853	3.0866	-1.6793	3.23119	0.00123275	0.0344297	0.31224275
XLOC_022694	SLC39A4	chr8:145637797-145642273	14.404	4.4748	-1.6866	3.45674	0.000546754	0.0191831	0.3106668
XLOC_004508	ALG8	chr11:77811987-77850699	73.991	22.9826	-1.6868	4.51454	6.35E-06	0.00059019	0.31061297
XLOC_024005	KIF4A	chrX:69509878-69640774	14.555	4.49137	-1.6963	4.0541	5.03E-05	0.00303383	0.3085829
XLOC_017397	FAT1	chr4:187508936-187644987	5.244	1.61368	-1.7003	4.13658	3.53E-05	0.00227154	0.30771784
XLOC_018036	PLPP1	chr5:54603575-54830873	122.96	37.7543	-1.7035	6.53007	6.57E-11	3.09E-08	0.30704883
XLOC_010286	NDC80	chr18:2571509-2616634	15.843	4.82241	-1.716	3.62266	0.000291588	0.0124449	0.30438307
XLOC_000992	FAM72A	chr1:206138910-206155074	11.603	3.49849	-1.7297	3.15879	0.00158424	0.0415454	0.3015251
XLOC_010624	-	chr18:75704080-75705667	23.984	7.22648	-1.7307	3.53681	0.000404989	0.0155968	0.30130573
XLOC_002079	KIF14	chr1:200520624-200589862	4.3878	1.31774	-1.7354	3.5481	0.000388028	0.0151526	0.30032365
XLOC_010618	-	chr18:75686261-75688763	63.735	18.9415	-1.7505	4.52253	6.11E-06	0.00057927	0.29719052
XLOC_003601	FAM111B	chr11:58874657-58894888	14.165	4.20696	-1.7515	3.64928	0.000262975	0.011523	0.29700312
XLOC_009818	TOP2A	chr17:38544772-38574202	55.092	16.3521	-1.7524	4.33728	1.44E-05	0.00114698	0.29681584
XLOC_001893	IQGAP3	chr1:156495196-156542396	11.877	3.50097	-1.7624	4.27918	1.88E-05	0.00139053	0.29476763
XLOC_013319	BUB1	chr2:111395408-111435684	16.176	4.76735	-1.7626	4.11663	3.84E-05	0.00241422	0.29472472
XLOC_014309	PMEPA1	chr20:56223451-56286541	59.202	17.4245	-1.7645	4.88583	1.03E-06	0.00014506	0.29432255
XLOC_009625	AURKB	chr17:8108048-8113883	13.538	3.98064	-1.7659	3.15116	0.00162622	0.0424151	0.29403504
XLOC_000839	NUF2	chr1:163291722-163325553	26.831	7.88136	-1.7674	4.41044	1.03E-05	0.00086596	0.29374577
XLOC_001024	DTL	chr1:212113740-212278187	30.64	8.99102	-1.7688	4.59531	4.32E-06	0.0004532	0.29344662
XLOC_005297	PUS1	chr12:132413744-132428406	10.652	3.10786	-1.7771	3.19887	0.00137968	0.0374718	0.29176121
XLOC_001587	DEPDC1	chr1:68939834-68962799	11.204	3.26522	-1.7788	4.91894	8.70E-07	0.00012804	0.2914257
XLOC_023316	ARHGEF39	chr9:35658286-35665278	8.5579	2.46216	-1.7973	3.56141	0.000368872	0.0146641	0.28770656
XLOC_022541	TMEM64	chr8:91634222-91658133	10.72	3.07296	-1.8025	4.43189	9.34E-06	0.00080785	0.28666944
XLOC_004978	ESPL1	chr12:53662082-53687427	9.5621	2.71728	-1.8152	4.32275	1.54E-05	0.00121538	0.28417273
XLOC_007523	TICRR	chr15:90118817-90198682	9.048	2.55984	-1.8215	4.31542	1.59E-05	0.00124446	0.28291881
XLOC_007255	ARHGAP11A	chr15:32907690-32931868	28.15	7.89455	-1.8342	5.78723	7.16E-09	1.91E-06	0.28044699
XLOC_001037	CENPF	chr1:214776531-214837914	62.101	17.4156	-1.8342	4.40002	1.08E-05	0.00089677	0.28043921
XLOC_018857	SLC16A10	chr6:111408780-111544606	8.3981	2.34808	-1.8386	3.39808	0.000678609	0.022451	0.27959879
XLOC_020866	ANLN	chr7:36363758-36493400	56.923	15.9115	-1.8389	4.76529	1.89E-06	0.00022134	0.27952709
XLOC_017550	CCNB1	chr5:68462912-68474070	34.442	9.58017	-1.846	4.36868	1.25E-05	0.00102218	0.27815482
XLOC_013922	FAM83D	chr20:37554954-37581703	7.7086	2.13234	-1.854	3.29254	0.000992851	0.0297523	0.27661667
XLOC_016546	TACC3	chr4:1723265-1746897	27.38	7.45941	-1.876	4.40943	1.04E-05	0.00086596	0.2724418

XLOC_017843	HMMR	chr5:162887516-162918953	48.281	13.1302	-1.8786	7.30456	2.78E-13	1.96E-10	0.27195314
XLOC_000619	FAM72B	chr1:120839004-120855681	10.243	2.73258	-1.9063	3.4792	0.000502911	0.0181619	0.26676846
XLOC_002070	ASPM	chr1:197053256-197115824	22.541	5.91177	-1.9309	4.85256	1.22E-06	0.00016238	0.26227096
XLOC_018045	DEPDC1B	chr5:59892738-59995993	8.2446	2.13904	-1.9465	3.38985	0.000699315	0.0230587	0.25944668
XLOC_016622	NCAPG	chr4:17812524-18023483	23.253	5.97893	-1.9595	4.77547	1.79E-06	0.00021296	0.25712112
XLOC_007250	ARHGAP11B	chr15:30918878-30931013	22.991	5.88071	-1.967	3.70424	0.000212022	0.00967745	0.25578614
XLOC_005350	CDCA3	chr12:6957971-6960456	23.651	5.92186	-1.9978	3.91851	8.91E-05	0.0048028	0.25038499
XLOC_014302	AURKA	chr20:54944444-54967351	25.951	6.39466	-2.0209	5.11486	3.14E-07	5.43E-05	0.24641295
XLOC_017446	TRIP13	chr5:892968-918164	10.227	2.51529	-2.0236	3.70003	0.000215575	0.00970544	0.24594199
XLOC_001720	ATP1A1-AS1	chr1:116915794-116961244	77.487	18.9963	-2.0282	3.77506	0.000159969	0.00769336	0.24515396
XLOC_002560	CDK1	chr10:62538088-62554610	12.756	3.08779	-2.0466	3.78166	0.000155787	0.00752895	0.24206057
XLOC_017723	KIF20A	chr5:137514416-137549032	20.964	5.07099	-2.0476	4.86278	1.16E-06	0.00015632	0.24188614
XLOC_010384	SKA1	chr18:47901391-47920538	16.297	3.93583	-2.0498	4.57784	4.70E-06	0.00048248	0.24151087
XLOC_017233	CENPE	chr4:104026962-104119566	8.5442	2.01317	-2.0855	4.83499	1.33E-06	0.0001683	0.23561936
XLOC_008138	PLK1	chr16:23690200-23724821	24.367	5.39771	-2.1745	4.73109	2.23E-06	0.0002502	0.22151557
XLOC_017199	ABCG2	chr4:89011415-89080011	3.2101	0.703955	-2.1891	3.34609	0.000819602	0.0258989	0.21929579
XLOC_000335	CDC20	chr1:43824625-43828873	32.817	6.87122	-2.2558	5.8357	5.36E-09	1.51E-06	0.20938355
XLOC_007367	CCNB2	chr15:59397283-59417244	31.622	6.57237	-2.2665	4.89409	9.88E-07	0.00014111	0.20784069
XLOC_006474	CDKN3	chr14:54863672-54886934	27.1	5.58324	-2.2791	4.05346	5.05E-05	0.00303383	0.20602053
XLOC_003303	-	chr10:96369043-96370107	19.462	3.86907	-2.3306	3.51252	0.000443878	0.0166395	0.1988028
XLOC_004298	SLC43A3	chr11:57174426-57195053	44.661	8.547	-2.3855	6.28178	3.35E-10	1.22E-07	0.19137576
XLOC_012319	CENPA	chr2:27008881-27017455	7.9309	1.23926	-2.678	3.18054	0.00147003	0.039264	0.1562567

LNCaP-AI

gene_id	gene	locus	LNCaP AI- siC (FPKM)	LNCaP AI- siAR (FPKM)	log2(fold_change)	test_stat	p_value	q_value	Fold Change siAR/siC
XLOC_016326	SI	chr3:164696685-164796283	1.83502	7.89118	2.10444	-7.35528	1.91E-13	1.92E-10	4.300308
XLOC_019638	-	chr6:160697871-160699952	6.15731	20.1266	1.70873	-5.96677	2.42E-09	1.11E-06	3.2687295
XLOC_000481	CLCA2	chr1:86889768-86922240	0.977479	3.04804	1.64074	-3.56068	0.0003699	0.0366344	3.1182574
XLOC_001317	CAMK2N1	chr1:20808883-20812728	11.5116	31.1463	1.43598	-6.20486	5.47E-10	2.63E-07	2.705659
XLOC_022462	CYP7A1	chr8:59402736-59412720	2.87231	7.67036	1.41708	-4.44409	8.83E-06	0.0016212	2.6704447
XLOC_003124	SORBS1	chr10:97071529-97321171	2.22796	4.81493	1.11179	-4.01858	5.86E-05	0.0079929	2.1611362
XLOC_004871	KIAA1467	chr12:13197314-13236383	11.3172	23.8247	1.07394	-5.45291	4.96E-08	1.76E-05	2.1051748
XLOC_023511	TNFSF15	chr9:117546914-117568408	1.85969	3.87822	1.06033	-3.64647	0.00026587	0.027977	2.0854085
XLOC_021999	FZD3	chr8:28351721-28431785	2.28868	4.66739	1.0281	-4.74684	2.07E-06	0.0004744	2.0393367
XLOC_016332	PDCD10	chr3:167401694-167452651	10.757	21.4518	0.99583	-3.76978	0.00016339	0.0201288	1.994222
XLOC_017701	MIR4461	chr5:134240809-134298336	4632.79	9041.85	0.96474	-5.44992	5.04E-08	1.76E-05	1.9517091
XLOC_009698	PMP22	chr17:15133095-15168644	17.9965	33.7334	0.90646	-4.04472	5.24E-05	0.0073501	1.8744366
XLOC_017262	SLC7A11	chr4:138948576-139163503	8.05256	14.1469	0.81296	-4.40001	1.08E-05	0.0018853	1.7568159
XLOC_022987	TMEM38B	chr9:108456824-108537444	24.6073	42.0879	0.77432	-3.72034	0.00019896	0.0231017	1.7103837
XLOC_017669	SLC12A2	chr5:127419482-127525380	17.3049	29.1327	0.75146	-4.11216	3.92E-05	0.0057387	1.6834922
XLOC_021605	ASNS	chr7:97481428-97501854	44.5532	74.23	0.73647	-6.42793	1.29E-10	7.69E-08	1.6660989
XLOC_018174	SKP1	chr5:133492081-133512724	21.4994	35.6891	0.73118	-3.46088	0.0005384	0.049445	1.6599997
XLOC_016328	BCHE	chr3:165490691-165555253	22.1285	36.3173	0.71475	-4.9084	9.18E-07	0.0002319	1.6412022
XLOC_015542	ALCAM	chr3:105085556-105295757	20.9419	33.6803	0.68551	-5.83368	5.42E-09	2.38E-06	1.6082738
XLOC_012596	GCC2	chr2:109065576-109125854	6.4038	10.2308	0.67592	-3.81328	0.00013713	0.0171027	1.5976119
XLOC_013433	GALNT3	chr2:166604312-166650803	25.4268	40.4819	0.67093	-3.57745	0.00034696	0.0350499	1.5920956
XLOC_008834	NQO1	chr16:69743303-69760533	35.2323	53.1125	0.59216	-3.6143	0.00030116	0.03073	1.5074969
XLOC_003448	NUCB2	chr11:17298285-17356105	40.4657	60.972	0.59145	-4.40576	1.05E-05	0.0018682	1.5067562
XLOC_012849	MAP2	chr2:210288770-210598834	10.0445	14.4636	0.52602	-3.91261	9.13E-05	0.0116754	1.4399473
XLOC_016281	HLTF	chr3:148747903-148804341	15.5264	22.2697	0.52036	-3.46815	0.00052406	0.0485692	1.4343121
XLOC_000419	NFIA	chr1:61542945-61928460	20.8903	28.5145	0.44887	-7.03403	2.01E-12	1.45E-09	1.3649669
XLOC_010544	DSC2	chr18:28644597-28682388	27.4468	36.9484	0.42887	-3.48418	0.00049365	0.0461748	1.3461816
XLOC_004271	FOLH1	chr11:49166111-49230222	381.004	496.694	0.38255	-4.08624	4.38E-05	0.0063269	1.3036431
XLOC_014544	APP	chr21:27252860-27543446	47.1587	61.1747	0.37541	-4.49444	6.98E-06	0.0013049	1.29721
XLOC_005511	SLC38A1	chr12:46576840-46663208	58.9207	75.8828	0.365	-3.98061	6.87E-05	0.009137	1.2878798
XLOC_016676	UGT2B10	chr4:69681712-69698534	77.7245	96.6258	0.31404	-3.9648	7.35E-05	0.0095137	1.2431833
XLOC_022578	YWHAZ	chr8:101930803-101965623	152.518	126.478	-0.27009	5.39385	6.90E-08	2.18E-05	0.8292667
XLOC_022894	VPS13A	chr9:79791671-80033576	32.1105	26.5405	-0.27485	5.27326	1.34E-07	3.76E-05	0.8265391
XLOC_010927	UBA52	chr19:18682613-18688270	82.7675	67.6622	-0.29072	3.49128	0.00048072	0.0453851	0.8174968
XLOC_016053	PBRM1	chr3:52579054-52719866	34.137	26.5935	-0.36026	5.56224	2.66E-08	1.03E-05	0.7790242
XLOC_002753	ADD3	chr10:111705316-111895323	259.807	202.073	-0.36256	5.29699	1.18E-07	3.40E-05	0.7777821
XLOC_004011	APLP2	chr11:129939715-130014706	89.3114	68.7236	-0.37804	3.66142	0.00025082	0.027825	0.7694828
XLOC_015902	THRB	chr3:24158644-24536313	26.5925	20.3339	-0.38713	4.31394	1.60E-05	0.0027001	0.7646487
XLOC_009036	CHD3	chr17:7788122-7816075	23.4835	17.9503	-0.38764	4.56389	5.02E-06	0.0009804	0.7643779
XLOC_010628	NETO1	chr18:70409548-70534810	117.068	88.4076	-0.4051	4.55705	5.19E-06	0.0009888	0.7551829
XLOC_009705	NCOR1	chr17:15933407-16118874	40.4487	30.5302	-0.40585	4.99858	5.78E-07	0.0001496	0.7547894

XLOC_022440	PRKDC	chr8:48685668-48872743	57.474	43.1783	-0.4126	9.16953	0	0	0.7512672
XLOC_015468	FLNB	chr3:57994126-58157982	68.279	48.2626	-0.50054	8.17651	2.22E-16	2.80E-13	0.7068436
XLOC_015805	LPP	chr3:187868993-188608460	11.5889	8.17101	-0.50415	3.72214	0.00019754	0.0231017	0.7050742
XLOC_012852	CPS1	chr2:211342405-211543831	15.1184	10.5309	-0.52168	3.66657	0.00024582	0.0275924	0.6965617
XLOC_005186	TXNRD1	chr12:104609558-104744062	37.1456	25.8681	-0.52202	4.70174	2.58E-06	0.0005791	0.6963971
XLOC_022467	ASPH	chr8:62200524-62627199	399.74	275.269	-0.53822	12.5995	0	0	0.6886205
XLOC_018311	FBXW11	chr5:171288555-171433877	28.2593	19.2599	-0.55313	3.96967	7.20E-05	0.0094426	0.6815408
XLOC_016632	LIMCH1	chr4:41362793-41702083	85.0746	57.4826	-0.56561	4.02282	5.75E-05	0.0079579	0.675672
XLOC_000836	UHMK1	chr1:162466963-162499419	37.1809	24.9031	-0.57824	7.07807	1.46E-12	1.14E-09	0.6697808
XLOC_005089	RASSF3	chr12:65004292-65091347	51.2883	33.0843	-0.63248	5.4118	6.24E-08	2.10E-05	0.6450652
XLOC_002607	ZMIZ1	chr10:80828791-81076285	25.9512	16.4417	-0.65844	3.63125	0.00028205	0.0290741	0.6335612
XLOC_019443	CDK19	chr6:110931180-111136412	27.2812	17.2671	-0.65988	3.65309	0.00025911	0.0278457	0.6329296
XLOC_013182	AAK1	chr2:69685126-69870977	11.7012	7.3612	-0.66864	3.74222	0.0001824	0.0219357	0.6290999
XLOC_000435	AK4	chr1:65613231-65697828	35.304	22.1361	-0.67344	3.64894	0.00026332	0.027977	0.627012
XLOC_003604	FAM111B	chr11:58874657-58894888	24.8815	15.5412	-0.67898	3.65584	0.00025634	0.0278445	0.6246089
XLOC_002139	C1orf116	chr1:207191865-207206101	46.7109	28.9776	-0.68882	7.21566	5.37E-13	4.93E-10	0.6203602
XLOC_007550	ABHD2	chr15:89631380-89745591	84.6532	52.1582	-0.69867	3.65879	0.00025341	0.027825	0.61614
XLOC_000731	IL6R	chr1:154377668-154441926	10.3916	6.35013	-0.71056	3.55522	0.00037766	0.0366835	0.6110816
XLOC_003075	KCNMA1	chr10:78629358-79397577	16.5388	10.0898	-0.71296	4.41128	1.03E-05	0.0018537	0.6100689
XLOC_005484	CAPRIN2	chr12:30862485-30907448	53.1294	32.2956	-0.71817	9.87149	0	0	0.607868
XLOC_022909	CTSL1	chr9:90340973-90346384	42.6594	25.7796	-0.72663	3.85726	0.00011467	0.0144796	0.6043126
XLOC_017492	GHR	chr5:42423876-42721980	28.0892	16.9709	-0.72695	5.40311	6.55E-08	2.13E-05	0.6041799
XLOC_013370	SMPD4	chr2:130908964-130948300	11.436	6.82593	-0.74449	3.57083	0.00035585	0.0355916	0.5968797
XLOC_000470	PTGFR	chr1:78956727-79006386	47.4373	28.1385	-0.75348	4.60846	4.06E-06	0.0008346	0.5931718
XLOC_010622	DSEL	chr18:65173818-65566856	16.8242	9.72319	-0.79104	4.20486	2.61E-05	0.0039987	0.5779285
XLOC_024858	CD24	chrY:21094584-21239302	45.7474	26.1835	-0.80503	4.25352	2.10E-05	0.0033744	0.5723494
XLOC_015709	ARHGEF26	chr3:153839148-153975616	28.5534	16.3154	-0.80743	4.27574	1.91E-05	0.003104	0.5713988
XLOC_001058	CAPN2	chr1:223889294-223963720	22.0359	12.5323	-0.8142	5.33905	9.34E-08	2.86E-05	0.5687242
XLOC_000970	PPP1R12B	chr1:202317829-202557697	11.7341	6.64679	-0.81998	6.21683	5.07E-10	2.56E-07	0.5664502
XLOC_019339	ELOVL5	chr6:53132195-53213977	46.3072	25.9912	-0.83321	4.60469	4.13E-06	0.0008346	0.5612778
XLOC_000918	DHX9	chr1:182808438-182857117	39.2453	21.8457	-0.84517	5.55334	2.80E-08	1.05E-05	0.5566452
XLOC_022111	ZBTB10	chr8:81398447-81434610	7.96184	4.42339	-0.84795	3.68532	0.00022842	0.0259559	0.5555744
XLOC_001533	RAB3B	chr1:52373627-52456436	12.7953	7.10037	-0.84965	4.61133	4.00E-06	0.0008346	0.5549213
XLOC_007456	BBS4	chr15:72978525-73030817	20.6987	11.3283	-0.8696	4.08209	4.46E-05	0.0063504	0.5472982
XLOC_016127	CLDND1	chr3:98234316-98241910	19.5344	10.633	-0.87747	3.75959	0.00017019	0.020714	0.5443219
XLOC_010534	ANKRD29	chr18:21179977-21242849	18.3215	9.96867	-0.87806	4.2449	2.19E-05	0.0034519	0.5440978
XLOC_012083	C19orf48	chr19:51300960-51307974	26.001	14.1434	-0.87844	3.53077	0.00041435	0.0394881	0.5439557
XLOC_002660	PCGF5	chr10:92980368-93044021	8.55307	4.59025	-0.89787	4.21301	2.52E-05	0.0039163	0.5366785
XLOC_017536	PIK3R1	chr5:67511583-67597649	23.3409	12.4552	-0.90611	6.38771	1.68E-10	9.45E-08	0.5336209
XLOC_023185	EHMT1	chr9:140513443-140730578	6.78428	3.59971	-0.91431	3.63711	0.00027572	0.0287144	0.5305961
XLOC_008857	ZFHX3	chr16:72816785-73092534	28.2435	14.9709	-0.91576	10.3512	0	0	0.5300646
XLOC_006866	SEC23A	chr14:39501122-39572437	11.6604	6.00215	-0.95807	4.11501	3.87E-05	0.0057387	0.5147458
XLOC_017102	UGT2B17	chr4:69402902-69434245	279.36	142.303	-0.97316	5.80724	6.35E-09	2.67E-06	0.5093877

XLOC_022892	PCA3	chr9:79226236-79521003	7.74449	3.84946	-1.00851	3.73552	0.0001873<br>3	0.022263<br>3	0.4970593
XLOC_023973	EDA	chrX:68835910-69259321	8.1186	4.00364	-1.01992	3.68503	0.0002286<br>8	0.025955<br>9	0.4931437
XLOC_010533	NPC1	chr18:21083461-21166581	34.9151	17.0579	-1.03342	5.60728	2.06E-08	8.30E-06	0.4885506
XLOC_002188	LBR	chr1:225589203-225616557	9.1403	4.34272	-1.07364	4.11571	3.86E-05	0.005738<br>7	0.4751187
XLOC_018024	PPAP2A	chr5:54603575-54830873	117.059	55.5873	-1.07441	6.8446	7.67E-12	5.16E-09	0.4748652
XLOC_003306	-	chr10:98750172-98751918	14.0507	6.44912	-1.12346	3.55563	0.0003770<br>8	0.036683<br>5	0.4589917
XLOC_012839	ADAM23	chr2:207308367-207482679	13.1584	5.98651	-1.1362	4.56284	5.05E-06	0.000980<br>4	0.4549563
XLOC_002439	VIM	chr10:17270257-17279592	14.1494	6.10528	-1.21261	4.35514	1.33E-05	0.002276<br>9	0.4314873
XLOC_014238	MAFB	chr20:39314516-39317876	37.7528	16.0296	-1.23584	6.47986	9.18E-11	5.80E-08	0.4245952
XLOC_021520	SPDYE7P	chr7:72333317-72339655	5.94509	2.48898	-1.25614	4.83236	1.35E-06	0.000326<br>7	0.4186626
XLOC_013504	TMEFF2	chr2:192814746-193059644	61.9498	25.6797	-1.27047	6.35868	2.03E-10	1.08E-07	0.4145247
XLOC_017642	KCNN2	chr5:113698015-113832197	13.069	5.32879	-1.29427	4.2806	1.86E-05	0.003086<br>7	0.4077424
XLOC_023806	REPS2	chrX:16964813-17171403	3.32948	1.32352	-1.33091	4.68482	2.80E-06	0.000615<br>3	0.3975174
XLOC_012090	KLK4	chr19:51409607-51413994	57.2401	22.1767	-1.36798	7.47603	7.66E-14	8.60E-11	0.3874333
XLOC_023421	BICD2	chr9:95473644-95527083	5.39743	2.04388	-1.40096	4.83101	1.36E-06	0.000326<br>7	0.3786771
XLOC_014314	PMEPA1	chr20:56223451-56286541	7.85443	2.9084	-1.43328	5.07421	3.89E-07	0.000103<br>4	0.3702881
XLOC_002127	SLC45A3	chr1:205626980-205649630	7.91518	2.88443	-1.45634	4.65091	3.30E-06	0.000710<br>3	0.3644165
XLOC_005482	TMTC1	chr12:29653745-29937692	3.81791	1.25732	-1.60242	5.33197	9.72E-08	2.89E-05	0.3293241
XLOC_013422	DPP4	chr2:162848754-162931052	3.69936	1.1462	-1.69042	3.98863	6.65E-05	0.008951<br>1	0.3098367
XLOC_023968	AR	chrX:66763873-66947868	97.6423	29.5328	-1.72519	14.667	0	0	0.3024587
XLOC_022372	NKX3-1	chr8:23536205-23540450	94.62	27.9191	-1.76089	9.52859	0	0	0.2950661
XLOC_024751	3' UTR AR	chrX:66948348-66950457	119.336	32.6211	-1.87115	10.0171	0	0	0.2733554
XLOC_024740	-	chrX:45639573-45640947	8.2172	1.9483	-2.07643	3.54006	0.0004000<br>4	0.038487<br>7	0.2371004
XLOC_011280	KLK3	chr19:51358170-51364020	22.2285	5.1287	-2.11575	5.22624	1.73E-07	4.72E-05	0.2307256
XLOC_024743	c19orf48	chr19:51300960-51307974	16.4318	3.71794	-2.14391	4.803	1.56E-06	0.000367<br>2	0.2262657
XLOC_011281	KLK2	chr19:51376688-51383823	10.9411	1.73963	-2.6529	7.0806	1.44E-12	1.14E-09	0.1590001
