# Supplementary material for: Alteration in expression and subcellular localization of the androgen receptor- regulated FAM111A protease is associated with emergence of castration resistant prostate cancer
Source: Neoplasia. 2025 May 29;66:101181. doi: 10.1016/j.neo.2025.101181 (PMC12159915; doi:10.1016/j.neo.2025.101181)
Supplement: Supplementary file 2 [file mmc2.pdf]

Alterations in FAM111A subcellular localization and expression during prostate cancer progression
SUPPLEMENTARY DATA 1

Figure 1. FAM111A sequence containing AR site

CTTGATTAGAAATGATCTCAAAACCTTTTAGAATTTCCAAAATCTTCATATTACFigureTGAAACTGTCGGAATATATGGGT
1F →→→→→→→→→→→→→→→→→→→→→→→→

CTTGAAATTCAGAAGATGATAGTCACTCTTCCCATATTTATAGGCTATTAAGGCAAGGGATATCTTAAACATCATAT
←←←←←←←←←←←←←←←←←←←←←←← 1R

TACTTTATTTAGATTTCTACTACTCCAATTATTAATGTTATGTATTTCTCATTGTTTTACTTCTTCATGGTATTATG

AAGACTATATAGATGATTCAACCAAGCCTGCAAATCTCCCTCTTGTGGAATTCCACTGGACCCAATCTGTTTTCCAT
2F →→→→→→→→→→→→→→→→→→→→→

TTCCATTGCAATACTACTAAAGCCATACAATATCAAGCACCCTCCCTCTAGGTCCAGGGACTATCACAGAAGAAGCA
←←←←←←←←←←←←←←←←←←←←← 2R

GGCATGTAAGATTTTAAGGACTGGTTTCGAGGGGTCGAGTGTAGGAAAACAGCCTGTTGCATTGTAAGAGTGATGTC
3F →→→→→→→→→→→→→→→→→→→→→

ATCTTGAAGAGCAGCTGGCATGATGACTGCTGTTTGACTCCTGCATACCAAGATATTCTGCAGCAATGTCTTTAAAC
←←←←←←←←←←←←←←←←←←←←← 3R 4F →→→→→→→→→→→→

AGTGCCGGTAGTACAGATAACCCCTCATAAAGATGCTTATCTAACCTCCCCAGTGTTCAGGTGTTTCACAAGAAAGT
→→→→→→→→→→→→

CTGAGATATGACTAGCTACACGTTTTGCCAAAAATGCTTGTTATATAAAGGGTACTTTTGGGAGGGTGAGTGCCGCC
←←←←←←←←←←←←←←←←←←←←← 4R

ATTTAGTGGCTGCTAGAAACATTGCTTCTGTTTGTAAGTTCCTATTAAATGTTTCTTTCTGAGAAACCGTATTTGTC

AGCCTCTTTCTTTGGCCTCTCAGCTTCTTTGGTCTTTGGGGGTAGGTTGGGGTAGGTTTGCATTGACCTGCTCACCG

TGAAACACCTGGTGACTTTTAATGAAGAATGGCATTAGAAACCAAGATCTAGGCACTAGATATGCTCATTGCTGGTG
5F →→→→

GCGTGTTGTTTCTTTTAGGCCCCCTCAGCTGACAGGGCAAAGAAATATTTGTGTGTATATTTACCCACATATGTAT
→→→→→→→→→→→→→→→→

ATGTACACATATGTATATATGCCTTGCTTATCTGTAAATTCCAACTCCAAACAGTTGGAAGCGTGATGCCTGCCATC
←←←←←←←←←←←←←←←←←←←←←←←←←←← 5R

CCTCACACATTCACTTAATTGCTTAATCCAAGTATACATATATAGCAGTGTCAGAATTGTTAACCTCTACCTCAATG

GGAAATAATTTTATCAACTAGAATACAGTGCCTATGTGCAATTTCCCTGCTTTTAGT

Figure 2. Primers used in ChIP study.

1ARfamF actgaaactgtcggaatatatgg

1ARfamR gatatcccttgccttaatagcc

2ARfamF ccactggacccaatctgttt

2ARfamR gacctagagggagggtgctt

3ARfamF gtttcgaggggtcgagtgta

3ARfamR atgcaggagtcaaacagcag

4ARfamF tgtctttaaacagtgccggtag

4ARfamR tcaccctcccaaaagtaccc

5ARfamF ggtggcgtgttgtttcttt

5ARfamR ggaatttacagataagcaaggcata

Alterations in FAM111A subcellular localization and expression during prostate cancer progression.
SUPPLEMENTARY DATA

Figure Primers used in RT PCR:

FAM111A F 5'-TCTGCTGTTTAAAAAGTATCCCTGT, FAM111A R 5'-TTCCGTGACCTCTGCTTCTT;

AR F 5'-GCCTTGCTCTCTAGCCTCAA, R 5'-GTCGTCCACGTGTAAGTTGC;

PSA F 5'- CACAGCCTGTTTCATCCTGA, R 5'- AGGTCCATGACCTTCACAGC;

TMPRSSE2 F 5'- GTGATGGTATTCACGGACTGG, R 5'- CAGCCCCATTGTTTTCTTGTA].

HPRT1 F 5'- GCCAGACTTTGTTGGATTTG , R 5'-CTCTCATCTTAGGCTTTGTATTTTG;
